# A phase II study of Bruton’s tyrosine kinase inhibition for the prevention of anaphylaxis

**DOI:** 10.1172/JCI172335

**Published:** 2023-08-15

**Authors:** Ragha V. Suresh, Collin Dunnam, Dhananjay Vaidya, Robert A. Wood, Bruce S. Bochner, Donald W. MacGlashan, Melanie C. Dispenza

**Affiliations:** 1 Division of Allergy and Clinical Immunology, Department of Medicine,; 2 Division of General Internal Medicine, Department of Medicine, and; 3 Division of Allergy, Immunology, and Rheumatology, Department of Pediatrics, Johns Hopkins University School of Medicine, Baltimore, Maryland, USA.; 4 Division of Allergy and Immunology, Department of Medicine, Northwestern University Feinberg School of Medicine, Chicago, Illinois, USA.

**Keywords:** Immunology, Allergy, Basophils, Mast cells

## Abstract

**BACKGROUND:**

IgE-mediated anaphylaxis is a potentially fatal systemic allergic reaction for which there are no currently FDA-approved preventative therapies. Bruton’s tyrosine kinase (BTK) is an essential enzyme for IgE-mediated signaling pathways and is an ideal pharmacologic target to prevent allergic reactions. In this open-label trial, we evaluated the safety and efficacy of acalabrutinib, a BTK inhibitor that is FDA approved to treat some B cell malignancies, in preventing clinical reactivity to peanut in adults with peanut allergy.

**METHODS:**

After undergoing graded oral peanut challenge to establish their baseline level of clinical reactivity, 10 patients had a 6-week rest period, then received 4 standard doses of 100 mg acalabrutinib twice daily and underwent repeat food challenge. The primary endpoint was the change in patients’ threshold dose of peanut protein to elicit an objective clinical reaction.

**RESULTS:**

At baseline, patients tolerated a median of 29 mg of peanut protein before objective clinical reaction. During subsequent food challenge on acalabrutinib, patients’ median tolerated dose significantly increased to 4,044 mg (range 444–4,044 mg). 7 patients tolerated the maximum protocol amount (4,044 mg) of peanut protein with no clinical reaction, and the other 3 patients’ peanut tolerance increased between 32- and 217-fold. 3 patients experienced a total of 4 adverse events that were considered to be possibly related to acalabrutinib; all events were transient and nonserious.

**CONCLUSION:**

Acalabrutinib pretreatment achieved clinically relevant increases in patients’ tolerance to their food allergen, thereby supporting the need for larger, placebo-controlled trials.

**TRIAL REGISTRATION:**

ClinicalTrials.gov NCT05038904

**FUNDING:**

AstraZeneca Pharmaceuticals, the Johns Hopkins Institute for Clinical and Translational Research, the Ludwig Family Foundation, and NIH grants AI143965 and AI106043.

## Graphical abstract

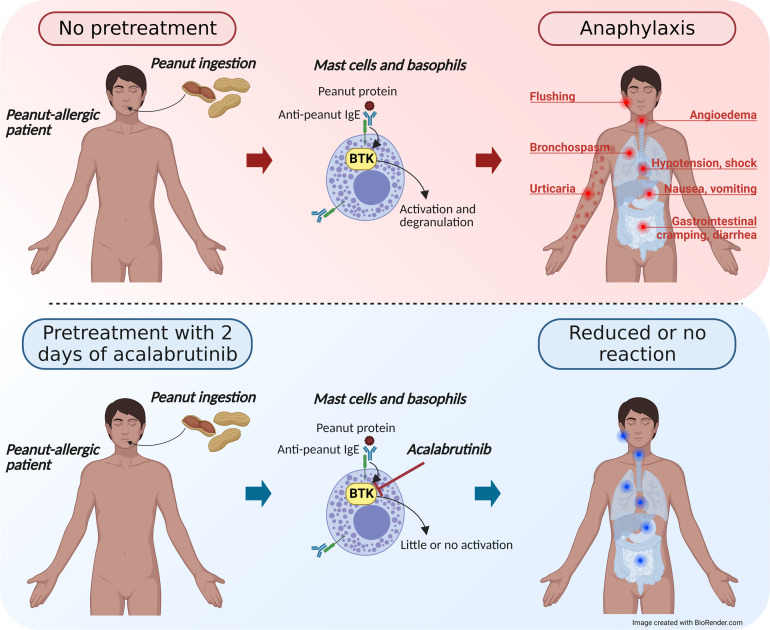

## Introduction

Anaphylaxis is an acute, potentially life-threatening systemic allergic reaction that may be caused by foods, medications, or stinging insect venom in allergic individuals (1). In IgE-mediated anaphylaxis, allergen cross-linking of specific IgE bound to the surface of mast cells and basophils initiates degranulation and release of mediators that cause urticaria, angioedema, bronchospasm, nausea, vomiting, diarrhea, hypotension, and/or shock (2). Although there are potential therapies under investigation, there are currently no approved therapies that can reliably prevent anaphylaxis (1). Though food oral immunotherapy products, including the FDA-approved peanut therapy Palforzia, can protect against accidental food exposures, they can also cause anaphylaxis from the food doses themselves. Intramuscular epinephrine administered after the onset of reaction is the only approved treatment that can potentially mitigate mortality from systemic reactions. Unfortunately, even with prompt and comprehensive medical treatment, anaphylaxis can still be fatal. Standard of care entails allergen avoidance, which is not always feasible for food or stinging insect allergies, where accidental exposures can occur. In addition, patients are often intentionally exposed to known allergens during diagnostic and therapeutic procedures including allergen skin testing, food and environmental allergen immunotherapy, and drug desensitizations, all of which carry the risk of a life-threatening reaction. Therefore, there is an unmet need for therapies that can prevent the occurrence and/or reduce the severity of anaphylaxis (3).

Acalabrutinib (Calquence; Acerta Pharma and AstraZeneca) is a second-generation oral, covalent inhibitor of Bruton’s tyrosine kinase (BTK), an essential enzyme for high-affinity IgE receptor (FcεRI) signaling in human mast cells and basophils (4–6). Acalabrutinib is currently FDA-approved for some B cell malignancies including chronic lymphocytic leukemia, small lymphocytic lymphoma, and mantle cell lymphoma, and is generally well-tolerated with chronic use (7, 8). In preclinical studies, pretreatment of primary human skin-derived mast cells and basophils with BTK inhibitors for 15 minutes completely prevented IgE-mediated cell activation, degranulation, and de novo cytokine production (9). Additionally, premedication with 2 oral human-equivalent doses of acalabrutinib just hours before allergen challenge abrogated moderate-severity anaphylaxis in a humanized mouse model and reduced mortality from severe anaphylaxis in the same model (9). Clinically, pretreatment of adults with 2 days (2 oral doses) of ibrutinib, a first-in-class BTK inhibitor, reduced or eliminated skin test reactivity to food allergens in food-allergic patients with no observed toxicities (10). Therefore, we hypothesized that BTK inhibitors would prevent clinical reactivity to food allergen ingestion in allergic patients. Here, we report the results of a phase II trial investigating the safety and efficacy of a 2-day course of acalabrutinib in preventing clinical reactivity to peanut in patients with peanut allergy.

## Results

### Trial design and patient characteristics.

**Figure 1:**
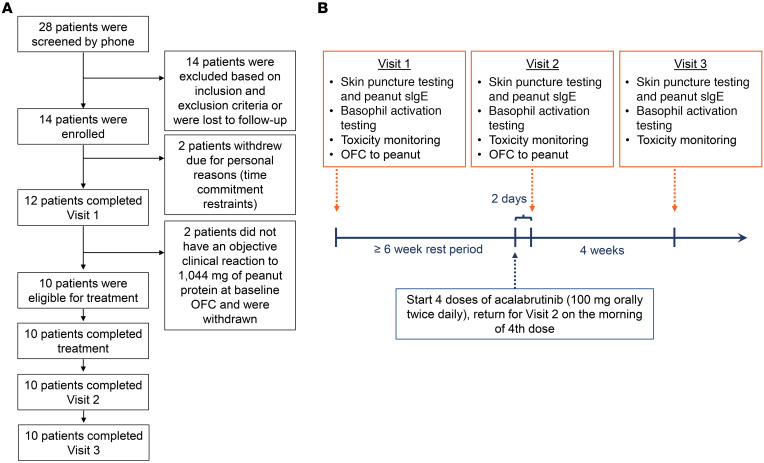
Schematic representing study design and patient disposition. Flow diagram summarizing the (**A**) enrollment and (**B**) study visit schedule for the trial. OFC, oral food challenge; sIgE, specific IgE.

This prospective, open-label trial enrolled adult patients with IgE-mediated peanut allergy (Figure 1A). Eligible patients underwent baseline placebo-controlled, single-blinded, graded oral food challenge (OFC; Supplemental Table 1; supplemental material available online with this article; https://doi.org/10.1172/JCI172335DS1) to determine their tolerant dose of peanut protein (Figure 1B). At the first sign of objective clinical reaction (as assessed by a predefined scoring system; Supplemental Table 2), the OFC was stopped and the reaction was treated. After their baseline OFC, patients underwent a rest period of at least 6 weeks before starting the study drug. All eligible patients then received 100 mg doses of acalabrutinib by mouth every 12 hours for a total of 4 doses, returning for repeat OFC on the morning of their last dose. Patients were required to stop all oral antihistamines for at least 7 days before study visits, but were permitted to use them and other allergy medications during the remainder of the study. Details of trial design, eligibility criteria, and study procedures are described in the Methods.

A total of 28 patients were screened, of whom 14 met eligibility criteria (Supplemental Table 3) and were enrolled (Figure 1A). Of the 14 patients, 2 withdrew from the study before the first visit due to personal scheduling conflicts. Twelve patients completed a baseline OFC to peanut, after which 2 patients were withdrawn because they failed to meet inclusion criteria for baseline clinical reactivity to peanut. The remaining 10 patients were eligible for treatment and completed the study (Supplemental Table 4). Baseline characteristics of the patients are shown in Table 1. Their mean age was 28 years (range, 23–36 years); 6 were female (60%), 4 were male (40%), 1 was African American (10%), 9 were white (90%), and 3 were Hispanic or Latino (30%).

### Primary endpoint.

**Figure 2:**
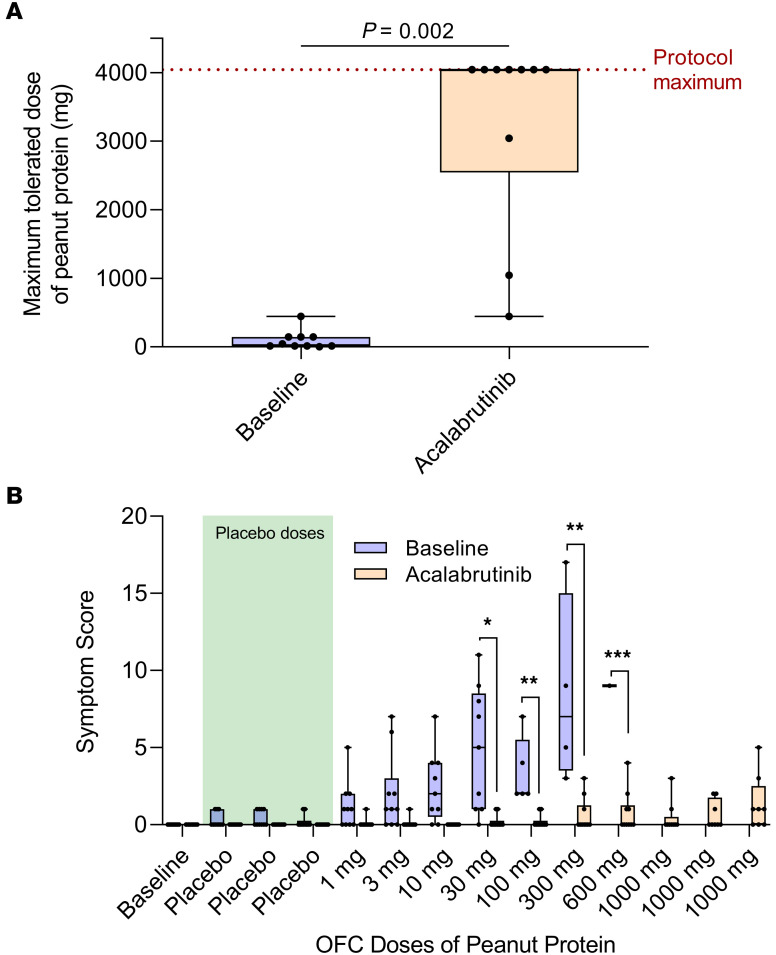
Maximum tolerated peanut dose and symptom scores during OFC. (**A**) The maximum tolerated dose of peanut protein at baseline (blue box) and during treatment with acalabrutinib (orange box) is shown for all patients (*n* = 10). The maximum protocol dose was 4,044 mg, thus, patients’ tolerated doses of at least 4,044 mg on acalabrutinib is plotted at this maximum. Data were tested using a Wilcoxon matched-pairs signed rank test. (**B**) Total symptom scores are shown during each placebo (benign food) and peanut dose during baseline OFC (blue boxes) and during OFC while on acalabrutinib (orange boxes). The shaded green area represents placebo doses of each OFC. Data were analyzed using 2-way ANOVA. All box plot midlines represent the median, boxes depict 25th and 75th percentiles, and whiskers depict range. *P < 0.05; **P < 0.01; ***P < 0.001. OFC, oral food challenge.

The predetermined primary endpoint was the change in patients’ threshold dose of ingested peanut protein to elicit an objective clinical reaction during OFC after acalabrutinib pretreatment compared with patients’ baseline. At baseline, patients tolerated a median of 29 mg of peanut protein (range, 1–444) before experiencing an objective clinical reaction during OFC. If needed, all patients were treated with intramuscular epinephrine for their reaction per standard of care in addition to adjunct therapies (e.g., antihistamines or albuterol) at the discretion of the investigator; a summary of rescue medications that were administered to patients during their baseline OFC is listed in Supplemental Table 5. During acalabrutinib treatment, patients’ median tolerated dose significantly increased to 4,044 mg (range, 444–4,044) of peanut protein (*P* = 0.002; Figure 2A). On acalabrutinib, 7 of 10 patients tolerated 4,044 mg, which was the maximum cumulative OFC peanut dose allowed in the study protocol, without having objective clinical reaction or requiring rescue medications. The remaining 3 patients’ tolerance increased from 14 mg of peanut protein at baseline to 444, 1,044, and 3,044 mg with acalabrutinib. These 3 patients who did not reach the maximum OFC peanut dose while taking acalabrutinib again received rescue medications, including intramuscular epinephrine, to treat their reactions. No patients had recurrent or second-phase reactions following OFC.

### Secondary endpoints.

A key secondary endpoint included the change in the severity of clinical reactions during OFC, as assessed by using a modified PRACTALL scale to score symptoms (11) (Supplemental Table 2). Symptom scores during OFC were significantly reduced during acalabrutinib therapy at several individual peanut doses compared with baseline OFC (Figure 2B). While on acalabrutinib, breakthrough symptoms for the 3 patients who did not reach the maximum amount of peanut included gastrointestinal symptoms (nausea, cramping, and/or diarrhea) in all 3 patients, and lower respiratory symptoms (cough and wheezing) in 1 patient. Of the 7 patients who tolerated the maximum food challenge dose, 5 experienced subjective symptoms during their OFC on acalabrutinib that were not protocol-defined dose-limiting reactions, including acid reflux (2 patients), mild nausea (1 patient), mild epigastric pain (1 patient), and scalp pruritus (1 patient).

**Figure 3:**
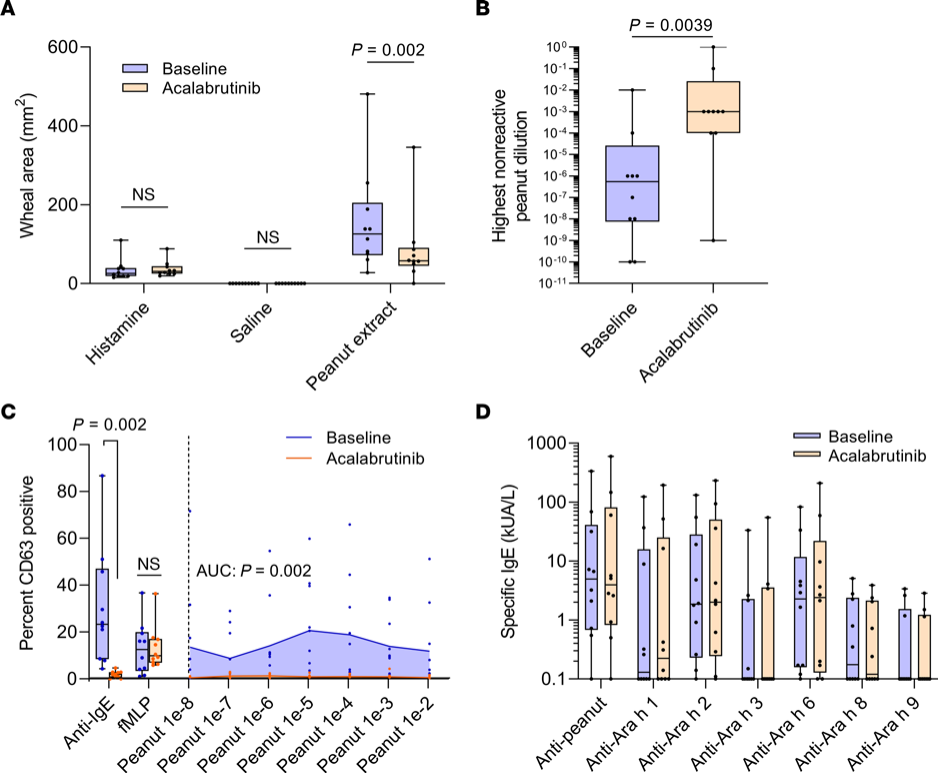
Secondary outcomes. (**A**) Skin puncture test wheal area (in mm^2^) to undiluted peanut extract and positive (histamine) and negative (saline) controls at patients’ baseline (blue boxes) and during treatment with acalabrutinib (orange boxes) are shown for all patients. Data were analyzed using Wilcoxon matched-pairs signed rank tests. (**B**) The highest concentration of peanut extract (original units, weight per volume) that produced a negative skin test at baseline and during acalabrutinib treatment is shown for all patients. Data were analyzed using Wilcoxon matched-pairs signed rank tests. (**C**) On the left side of the graph, the percent of basophils activated ex vivo in response to anti-IgE and fMLP are shown for all patients at baseline (blue boxes) and during treatment with acalabrutinib (orange boxes). On the right, basophil response percentages are displayed for each peanut extract dilution at baseline (blue AUC) and after acalabrutinib treatment (orange AUC). Data were analyzed using Wilcoxon matched pairs signed rank tests for each treatment or dilution. (**D**) Peanut and peanut-component specific IgE levels for all patients at baseline (blue boxes) and during acalabrutinib treatment (orange boxes) are shown. Data were analyzed using 2-way ANOVA. All graphs depict data from all patients who completed treatment (*n* = 10). All box plot midlines represent the median, boxes depict 25th and 75th percentiles, and whiskers depict range. fMLP, N-formylmethionyl-leucyl-phenylalanine.

Skin puncture testing to peanut extract was included as a secondary endpoint and surrogate marker of mast cell reactivity in vivo. All patients had a positive skin puncture test to undiluted peanut extract at baseline, with a median wheal area of 126 mm^2^ (range, 27.5–480). During treatment with acalabrutinib, skin test size to peanut extract was significantly reduced to a median of 57.7 mm^2^ (range, 0–345; *P* = 0.002; Figure 3A). The trend toward suppression of skin tests was observed at all dilutions of peanut extract (Supplemental Figure 1). When analyzing the highest peanut extract dilution to produce a negative skin test, it was observed that patients’ skin test ‘tolerance’ had increased by a mean of 3.4 ± SEM 0.92 log_10_ units (*P* = 0.0039; Figure 3B). Histamine and saline controls were unaffected by acalabrutinib. All skin tests had returned to baseline value sizes by the third study visit, which was 4 weeks after cessation of acalabrutinib (median 186 mm^2^, range 23.6–603; (Supplemental Figure 1). Though the median skin test size trended toward an increase at follow up compared to baseline, there was no significant difference between these timepoints, nor was there a difference detected in patients’ highest nonreactive peanut extract dilution between baseline and follow up.

An additional secondary endpoint was the percentage of basophils activated ex vivo by peanut extract. All patients had a positive basophil activation test at baseline to at least 1 dilution of peanut extract (mean peak response to peanut, 31.7%; range, 3.5–70.7; Figure 3C). While taking acalabrutinib, all patients had completely suppressed basophil activation at all peanut extract dilutions (mean peak response, 1.56%; range, 0–3.9; *P* = 0.002). Anti-IgE responses were also wholly suppressed with acalabrutinib treatment (mean 1.81%; range, 0–4.6) compared with baseline (mean, 30.1%; range, 4.3–86.7; *P* = 0.002). All basophil activation responses to anti-IgE antibody and peanut extract returned to baseline values by the third visit (anti-IgE mean, 27.1%, range, 4.5–51.1; peanut mean, 28.0%; range, 4.3–67.7). Ex vivo basophil activation by N-formylmethionyl-leucyl-phenylalanine (fMLP), a non-IgE-mediated stimulus, was unchanged by acalabrutinib therapy.

### Exploratory endpoints.

Because BTK plays an important role in B cell receptor signaling and therefore affects plasma B cell survival, exploratory endpoints included markers of humoral immunity function and allergy. Based on earlier studies (10), we hypothesized that the short duration of exposure to acalabrutinib would not affect either quantitative immunoglobulins or peanut-specific IgE in serum. All patients had a positive detectable specific IgE to peanut and/or at least 1 peanut component at baseline, and 9 out of 10 patients had specific IgEs to multiple peanut components (Figure 3D). No changes in specific IgEs were detected during acalabrutinib therapy or at follow up (Supplemental Figure 2) compared with patients’ baseline. Additionally, levels of quantitative immunoglobulins remained unchanged while on acalabrutinib and at follow up (Supplemental Table 6).

### Safety.

Safety endpoints included electrocardiography and laboratory blood testing, including complete blood counts and differentials, serum chemistries, and liver function tests. A total of 15 adverse events occurred in 5 of 10 patients (50%; Supplemental Table 7). Of 15 adverse events, 4 were deemed to be at least possibly related to acalabrutinib (summarized in Table 2), which included 1 grade-2 neutropenia (2.65 to 1.22 K/mm^3^), 1 grade-1 decrease in hemoglobin (by 0.3 g/dL), and 1 grade-1 peripheral eosinophilia (from baseline absolute eosinophil count of 250/mm^3^ to 890/mm^3^), which were observed at the second visit and resolved by the third visit. A single grade-1 increase in liver function tests was observed at the third visit and was not resolved at study completion because the patient was lost to follow up. For all adverse events possibly related to acalabrutinib, patients remained otherwise asymptomatic. No patients reported any symptoms while taking acalabrutinib, and none discontinued treatment due to side effects or toxicity. Mean laboratory values across all patients were unchanged with the exception of reduced hemoglobin (mean 13.3 ± 1.60 to 13.2 ± 1.44 g/dL) and increased absolute lymphocyte count (mean 1.99 ± 0.48 to 2.43 ± 0.40 K/mm^3^) while taking acalabrutinib, though both remained within normal laboratory range at all study visits (Supplemental Table 6).

Among adverse events not attributed to acalabrutinib, 1 patient experienced recurrent wheezing after her baseline OFC despite treatment with epinephrine and was sent to the emergency department for further management. Two sports-related concussions occurred in 2 patients during the trial that were considered to be unrelated to study drug or procedures; 1 occurred before the patient received acalabrutinib, and 1 occurred 18 days after the patient’s last dose of acalabrutinib. One of these same patients also experienced a separate mechanical fall due to tripping over an object, which occurred before acalabrutinib therapy. No deaths or treatment-related serious adverse events occurred during the trial. No electrocardiographic changes were observed during acalabrutinib treatment.

## Discussion

This trial has demonstrated the first-ever treatment to achieve rapid-onset prevention of IgE-induced food reactivity. Results showed that a short course of premedication with standard dosing of the BTK inhibitor acalabrutinib can achieve marked reduction or even complete elimination of clinical reactivity to ingestion of food allergens in allergic patients. Because tolerance to at least 300 mg of peanut protein is considered to be protective against reaction from an accidental exposure (12), all patients in this trial achieved clinically meaningful increases in their tolerance to peanut. Furthermore, 7 of 10 patients achieved tolerance to the maximum peanut dose of 4,044 mg of protein (about 16 to 20 peanuts), a tolerance level that has been shown to effectively eliminate all clinical reactivity to peanut exposures during oral immunotherapy. While foods are a very common cause of anaphylaxis (13, 14), regardless of the inducing allergen, all IgE-mediated anaphylaxis in humans is mediated by the FcεRI pathway, of which BTK is an essential kinase. We chose to include peanut-allergic patients in this trial because peanut is one of the most severe food allergies, and there is a strong precedent for utilizing graded OFCs in food allergy trials (13). Because of shared underlying mechanisms between cases of anaphylaxis, results herein are expected to be applicable for any IgE-mediated systemic allergic reaction.

In parallel with their clinical tolerance, patients’ highest negative skin test dilution of peanut also increased several logs. However, acalabrutinib treatment did not completely suppress skin tests as was observed for basophil activation, which was abolished by acalabrutinib treatment. This is in line with prior studies showing that BTK inhibitor doses that can suppress ex vivo basophil activation responses do not completely inhibit skin tests (10). The reasons for this disparate activity of BTK inhibitors on identical FcεRI pathways in mast cells versus basophils are as yet unknown. In vitro data suggest that the inhibitory concentrations of BTK inhibitors are similar in human mast cells and basophils (9). In a previous trial assessing ibrutinib’s effects on food allergen skin tests, 7 days of ibrutinib treatment did not suppress skin tests further than treatment for 2 days (10). Therefore, one could speculate that longer duration of BTK inhibitor therapy would not offer additional protection against anaphylaxis, and that skin mast cell inhibition in vivo is simply incomplete at the doses of irreversible BTK inhibitors that are FDA-approved for malignant indications. The dose of acalabrutinib utilized in this trial, which is the FDA-approved dose for treating B cell malignancies, has been shown to result in 100% drug occupancy of BTK in peripheral blood mononuclear cells (15, 16). However, the penetrance of BTK inhibitors into organs such as the skin has not been studied. Full inhibition of tissue-resident mast cells may require different BTK inhibitor dosing than what is used for treating cancers. Notably, the 3 patients who did not achieve full protection with acalabrutinib treatment did not demonstrate any symptoms of the skin or mucosa while on acalabrutinib as they had during their baseline OFC.

It is unknown why some patients did not achieve the same magnitude of clinical protection from food-induced anaphylaxis with acalabrutinib treatment as others. Interestingly, these results illustrate the role of mast cells in food-induced anaphylaxis, given that some patients still reacted to peanut ingestion despite complete inhibition of their basophils by acalabrutinib. No correlation was observed between patients’ clinical response and their baseline skin test size or percentage reduction; baseline basophil activation; specific IgE to peanut or components; total IgE or specific-to-total IgE ratios; baseline tryptase level (data not shown); body weight or body mass index, though our study was not powered to detect such correlations. Further trials are necessary to determine the minimum dose of BTK inhibitors required to attain reliable protection against systemic allergic reactions for all patients.

Given their remarkably rapid onset of action (within 2 days), BTK inhibitors may be a superior choice as adjunct prophylactic therapy for procedures such as high-risk immunotherapy or desensitizations compared with the anti-IgE monoclonal antibody omalizumab, which requires at least 4–8 weeks to achieve an effect and which does not reliably increase tolerance in every patient (17). Based on in vitro data and animal models, it may be possible to utilize inhibitors of essential kinases such as BTK or spleen tyrosine kinase (SYK) to prevent IgE-mediated reactivity during these procedures without interfering with the allergen desensitization process in mast cells and basophils (18, 19). For example, BTK inhibitors could be administered for 2 days at the onset of food oral immunotherapy to allow patients to rapidly reach their maintenance dose without adverse reactions and discontinued once allergen desensitization (hyporesponsiveness) is achieved in order to prevent interference with the long-term induction of protective allergen-specific IgG responses. Additionally, though this trial did not assess the recovery time of skin tests and basophil activation after cessation of acalabrutinib, prior human studies have shown that these parameters return to baseline within a week of cessation of covalent BTK inhibitor therapy (10), suggesting that these medications have a short duration of action, which may be advantageous in some clinical contexts. However, further trials will need to determine if this short duration of action would be sufficient to maintain clinical hyporesponsiveness in patients achieving high-dose food ingestion during oral immunotherapy.

Our results, in addition to prior trials utilizing the first-in-class BTK inhibitor ibrutinib to suppress allergen skin tests (10, 20), demonstrate that brief treatment with BTK inhibitors is well-tolerated in healthy patients. Laboratory changes in our study were mild to moderate in severity, largely reversible, and did not cause symptoms or illness in patients. In line with previous studies, neither quantitative immunoglobulins nor peanut-specific IgEs measured in serum were changed after a short course of a BTK inhibitor (10). To date, there are no data on the safety of chronic use (months to years) of acalabrutinib in healthy patients without cancers. The most common side effects of chronic acalabrutinib therapy in patients with cancer include gastrointestinal upset, headache, and infection, along with more rare but serious side effects including cytopenias, bleeding, arrhythmias, and hypertension (21). Interestingly, many of these side effects are thought to be due to off-target effects, and could theoretically be avoided by the use of compounds with higher specificity for BTK. Encouragingly, next-generation BTK inhibitors that are currently in development for chronic urticaria and autoimmune diseases are more selective for BTK with fewer off-target effects, and therefore show more favorable side effect profiles (22–25). For example, 12 weeks of remibrutinib (Novartis) was well-tolerated in a phase II trial for chronic urticaria with no observed bleeding, arrhythmia, or hypertension events in the treatment arms (22), as well as during the open-label extension of this trial for up to 52 weeks. Further studies are needed to delineate the safety and utility of prolonged administration of BTK inhibitors before these drugs could be used chronically in healthy patients for allergy indications, for example, to prevent reactivity from an accidental food exposure.

Limitations of our study include a small patient population, the lack of fully blinded OFCs, and the lack of a placebo treatment arm. We attempted to mitigate any potential placebo effects or bias by using a modified PRACTALL scale (11) to maintain objectivity when assessing symptoms and continuing each OFC until an objective clinical reaction, or the final peanut dose, was achieved. Additionally, this trial did not investigate alternative durations or dosages of acalabrutinib; therefore, the minimum effective duration and dose are as yet undetermined.

In conclusion, we have shown that pretreatment with the oral BTK inhibitor acalabrutinib for just 2 days significantly increases peanut-allergic patients’ tolerance to peanut during oral exposure. These results support the need for larger, placebo-controlled trials to further evaluate the safety and efficacy of these drugs in preventing IgE-mediated anaphylaxis. In particular, dose-finding trials will be necessary to determine the minimal effective dose of BTK inhibitors needed to prevent morbidity and/or mortality from either therapeutic or accidental allergen exposures before these medications can be used in clinical practice.

## Methods

### Study design.

The full trial protocol is available in Supplementary information. While the trial was partially supported by a research agreement from AstraZeneca, the design and conduct of the study were performed entirely by the investigators. All study procedures were conducted at a single site — Johns Hopkins University School of Medicine — in accordance with international ethics guidelines and local ethical and legal requirements, including the Declaration of Helsinki. Study visits were completed in the Clinical Research Unit at the Johns Hopkins University Bayview Campus in Baltimore, Maryland after written informed consent was obtained. All study visits occurred between December 2021 and October 2022. Patients stopped all antihistamines and medications with antihistamine properties at least 1 week before study visits in preparation for skin puncture testing and OFC. At Visit 1, medical history, vital signs, height, and weight were collected, and an electrocardiogram was performed (Figure 1). Patients underwent pregnancy testing (if applicable), skin testing, and basophil activation testing before food challenge. They then completed an OFC to peanut to confirm clinical reactivity at baseline. Patients with asthma underwent in-office spirometry before beginning OFC to confirm adequate asthma control, which was defined as having a forced expiratory volume in 1 second ≥ 80% of predicted for the patient. If they had an objective clinical reaction to a cumulative dose of 1,044 mg of peanut protein or less at baseline, patients were continued in the study. Visit 1 was followed by a rest period of at least 6 weeks. At the end of this rest period (and 2 days before visit 2), all patients began treatment with 4 standard oral doses of acalabrutinib (100 mg; AstraZeneca) every 12 hours. Patients received their fourth and final dose of acalabrutinib on the morning of visit 2. At visit 2, patients underwent the same procedures as visit 1. Four weeks following visit 2, all patients returned for a follow-up visit (visit 3) for repeat skin testing, basophil activation testing, and laboratory testing.

### Patient recruitment, screening, and eligibility.

Eligible patients were 18 years of age or older at screening with a history of an IgE-mediated allergy to peanut. Patients were required to have a positive skin puncture test to peanut extract and an objective clinical reaction to cumulative dose of 1,044 mg of peanut protein or less during baseline OFC. Key exclusion criteria included: cardiovascular disease or prior cerebrovascular accident; active infection; history of bleeding disorder or receiving anticoagulants; any immunomodulatory therapies or oral corticosteroids within 1 month before study participation; active infection or latent hepatitis; use of strong CYP3A4 inducers or inhibitors; and pregnancy or nursing. Patients were also excluded if they had ever received peanut immunotherapy or omalizumab. Complete eligibility criteria are listed in Supplemental Table 1. Patients who were taking proton pump inhibitors were instructed to stop these medications 7 days before enrollment.

Patients were recruited from the Johns Hopkins University Allergy and Clinical Immunology outpatient clinic and through IRB-approved advertising on social media. Patients who responded to advertisements were initially screened by telephone to determine eligibility. If determined eligible, patients were consented by teleconference before visit 1 in compliance with FDA 21 CFR Part 11.

### Medical history and demographics.

Age and information about medical comorbidities including food allergies and other atopic disorders were collected at intake. Patients were asked to report their biologic sex (options included male and female) and gender identity (options included male, female, unspecified, and prefer not to answer). Patients were also asked to report their race (options included American Indian or Alaskan Native, Asian, Black or African American, Native Hawaiian or other Pacific Islander, White, unknown, or not reported) and ethnicity (options included Hispanic or Latino, not Hispanic or Latino, unknown, and not reported).

### Endpoints.

The predetermined primary endpoint was the change in patients’ threshold dose of ingested peanut protein to elicit an objective clinical reaction during OFC after acalabrutinib pretreatment compared with the patients’ baseline. A key secondary endpoint included the change in the severity of clinical reactions during OFC. Other secondary endpoints included size of the skin test wheal to peanut extract and the percent of basophils activated ex vivo by peanut extract while receiving acalabrutinib compared with baseline. Safety endpoints included electrocardiography and laboratory blood testing, including complete blood counts and differentials, serum chemistries, and liver function tests. Exploratory endpoints included changes in circulating quantitative immunoglobulins and serum-specific IgE to peanut and peanut components.

### Skin puncture testing.

End-point titration skin puncture testing was performed using whole peanut extract (Greer), undiluted and in 9 serial 1:10 dilutions (original units given by manufacturer, weight/volume). Histamine (1 mg/mL; ALK) and saline (Greer) were used as positive and negative controls, respectively. Lincoln Diagnostics Multi-Test II devices and testing trays were used for skin testing application for all extract dilutions and controls. Skin tests were read 15 minutes after application. The wheal and flare were each circled with a ballpoint pen and transferred to a clear adhesive sheet, from which the largest diameter and its shortest perpendicular diameter for each wheal was measured in millimeters. Skin test wheal area was calculated as π × (average radius)^2^.

### Oral food challenge to peanut.

All patients underwent a patient-blinded, placebo-controlled, graded OFC to peanut at visit 1 to establish their baseline level of clinical reactivity. The food challenge protocol was designed to detect the “no observed adverse effect level”, or the highest dose observed not to produce any adverse effect, for each patient (26). While not ideal, it was essential that both placebo and peanut challenges be completed on the same day due to regulatory constraints on the duration of acalabrutinib dosing. To accomplish this, subjects were given 3 varying doses of placebo followed by graded doses of peanut (Supplemental Table 1). The amounts of placebo (oat flour; Bob’s Red Mill) or peanut (organic defatted light roast peanut flour; Anthony’s) needed for each dose were calculated based on the target protein amount and the protein content per weight listed on food packaging. Doses were prepared by mixing dry flour with chocolate pudding (Snack Pack) as a vehicle. Visit 1 began with placebo challenge and then immediately continued with peanut food challenge in increasing doses from 1 mg to 1,000 mg peanut protein, for a total cumulative goal amount of 4,044 mg of peanut protein. Patients were blinded to peanut or oat doses by using nose clips during dose consumption and coating their mouth with a flavored beverage such as juice or coffee immediately after each dose. Vital signs and physical exam were repeated roughly every 15 minutes throughout the OFC. Symptoms were assessed during OFC by a board-certified allergist/immunologist using a predefined symptom scale (Supplemental Table 2; further details can be found below under Symptom Score Assessment). Food doses were given every 15 minutes until a patient had an objective clinical reaction as determined by the symptom scoring scale, at which point the food challenge was stopped, and the reaction was treated using intramuscular epinephrine, plus additional adjunct therapies at the discretion of the investigator (Supplemental Table 5). In the event of moderate to severe subjective symptoms alone, the time between food challenge doses was extended, or the food challenge was stopped per the scoring system. All patients underwent identical repeat OFC at visit 2 to establish their new level of clinical reactivity while taking acalabrutinib.

### Symptom score assessment.

Adapted from the PRACTALL scale (11), a predefined scoring system was utilized to assess symptoms during OFC and determine clinical reactivity (Supplemental Table 2). Symptoms were color-coded to indicate the level of severity and likelihood of their representing true clinical reactivity rather than an anxiety reaction. In brief, green scores included symptoms that did not likely represent a true reaction and were not an indication to stop or delay peanut dosing. Orange scores were judged to be representative of a true reaction if 2 or more orange symptoms recurred for 3 consecutive peanut doses, in which case the food challenge was considered positive, and dosing was stopped. In the case of single, isolated orange symptoms, dosing was continued and/or delayed based on the clinical judgement of the principal investigator. Red scores represented objective symptoms that were highly likely to represent a true clinical reaction; therefore, any red symptom was an indication to stop dosing. Individual symptoms were recorded at each food dose, and combined symptom scores for each dose were calculated.

### Basophil activation testing.

Whole blood samples drawn into 4 mL lithium heparin phlebotomy tubes (BD Biosciences) before food challenge at each visit were utilized for basophil activation testing. Whole blood was incubated with mouse IgM anti-human-IgE monoclonal antibody (clone 6061P, Hybridoma Labs), the indicated dilutions of peanut extract (Greer), 1 μM fMLP (Sigma), or vehicle (Greer incipient control solution) in PAGCM buffer (piperazine-N,N′-bis[2-ethanesulfonic acid] + bovine serum albumin [MP Biomedicals] + glucose [Sigma-Aldrich] + 1.7 mM calcium + 1.7 mM magnesium) for 30 minutes at 37°C. Cells were then fixed using Phosflow Fix Buffer (BD Biosciences), centrifuged at 400*g* for 5 minutes, and resuspended in Pipes buffer with 1 mM EDTA and 0.25% BSA. Cells were blocked with 1 mg/mL nonspecific human IgG (MB Biological) and then incubated with the monoclonal antibodies anti-CD63 (1/1,000, BD-Pharmingen) and anti-FcεRIα (clone CRA-1, 1/250, Life Technologies) for 25 minutes at room temperature, then with secondary antibodies anti-CD123-PE (1/100, BD Biosciences), anti-mouse IgG2b-Alexa Fluor488 (1/1,000, Life Technologies), and anti-mouse IgG1-Alexa Fluor647 (1/1,000, Life Technologies) for 25 minutes at room temperature before analysis on a BD Accuri C6 flow cytometer. The percentage of CD63^+^ cells was recorded for each sample. All conditions were normalized by subtracting the percent activation in vehicle-treated samples; in the event of a negative value from this normalization, the result was recorded as zero. The highest percent of basophil activation obtained from 3 anti-IgE concentrations (0.1, 1, or 10 μg/mL) was reported as the maximum IgE-mediated stimulation for that sample.

### Laboratory testing and toxicity monitoring.

All patients underwent medical interview, physical exam, and laboratory testing at all visits to monitor for safety. Laboratory testing for toxicity monitoring (complete blood counts, serum chemistries, and quantitative immunoglobulins) was performed at each visit prior to OFC by the Johns Hopkins Core Pathology Laboratory. Adverse event determinations were made using the FDA Guidance for Industry: Toxicity Grading Scale for Healthy Adult and Adolescent Volunteers Enrolled in Preventive Vaccine Clinical Trials. Hepatitis serologies were also performed at visit 1. Before the start of the food challenges, laboratory quantification of total IgE and specific IgE to peanut and peanut components were performed using the Phadia ImmunoCAP platform by the Johns Hopkins Dermatology, Allergy, and Clinical Immunology Reference Laboratory. The lower limit of detection for the Phadia system is 0.1 KUA/L for all specific IgEs.

### Statistics.

All null hypothesis significance tests were 2-tailed. Normality testing was performed on all data using the Shapiro-Wilk test (α = 0.05). In the event that data did not demonstrate normal Gaussian distribution, nonparametric tests were utilized as described. For tests using multiple comparisons, corrections for multiplicity were employed as described, and only the *P*_adj_ values are presented in the manuscript. In analyses where there was a significant dose by treatment interaction at the *P* = 0.05 level, posthoc differences were presented separately for each dose using the Wilcoxon matched pairs signed rank test. All data were analyzed using Graphpad Prism software, version 9.2.0.

All patients who received acalabrutinib (*n* = 10) were included in the data analyses. A single blood sample was misplaced by the core pathology laboratory, resulting in missing data for Patient 006’s complete blood counts at visit 2; this was considered to have been a random occurrence, and sampling could not be repeated based on the timing of the sample draw. Basophil activation data were lost for Patient 008’s visit 3 due to cytometer malfunction; this was also considered to have been a random occurrence. Due to the randomness of these 2 individual events, no statistical adjustments were made.

Sample size was determined pretrial based on the primary outcome. It was estimated that 10 subjects allowed for 80% power to detect a 3-fold increase (1.1 natural log units; i.e. 1 food-dose escalation) in the threshold food dose using a paired *t* test with *P* < 0.05. For this sample size determination, the primary endpoint was assumed to be normally distributed with a SD of 1.1 natural log units.

Because most patients tolerated the highest cumulative amount of peanut during food challenge after acalabrutinib treatment, the primary outcome was analyzed as a censored variable after trial using a Wilcoxon matched-pairs signed rank test with *P* < 0.05.

For symptom scores, 2-way RM ANOVA with Geisser-Greenhouse correction was used to determine the interaction between treatment (baseline versus acalabrutinib), food challenge dose, and the patient on total symptom scores at each food challenge dose and an interaction effect of treatment by challenge dose. Once a patient displayed an objective clinical reaction, symptom scoring was ceased. For statistical analysis only, the final symptom score value was duplicated for the remainder of (unconsumed) food doses in order to perform ANOVA. This adjustment is not reflected in the graph in Figure 1, where symptom scores are not displayed after the final tolerated food dose. For multiple comparisons between baseline and acalabrutinib at each food challenge dose, Šídák’s multiple comparisons correction was used, with individual variances computed for each comparison, and an α threshold of 0.05.

Due to nonnormal distribution of skin puncture testing data, the significant interaction effect between treatment and extract dilution, and that many skin tests showed no wheal response at lower concentrations of peanut extract, skin test size to undiluted peanut extract was analyzed separately using a Wilcoxon matched-pairs signed rank test. The highest nonreactive skin test was also analyzed using a Wilcoxon matched-pairs signed rank test (*P* < 0.05) due to its nonnormal distribution.

Because the interaction effect between treatment and extract dilution was significant, and at lower concentrations of extract many basophil activation responses were 0, peanut extract dilution responses were analyzed using the AUC. The mean response for each patient across all peanut extract dilutions was calculated and analyzed using Wilcoxon matched-pairs signed rank test to compare baseline to acalabrutinib treatment. This mean height was not multiplied by width given that the peanut extract has only relative units (w/v). Peanut response means were graphed as AUC. This test was also used to compare responses to anti-IgE and fMLP.

To analyze peanut- and peanut component-specific IgE values, a 2-way RM ANOVA with Geisser-Greenhouse correction was used to determine interaction between treatment (baseline versus acalabrutinib), peanut (or component), and patient on the level of specific IgE. Multiplicity-*P*_adj_ values were calculated using a family wise alpha threshold of 0.05.

Other laboratory values obtained for safety analysis were analyzed with 1-way ANOVA with the Geisser-Greenhouse correction when applicable (for serum chemistries and quantitative immunoglobulins), and otherwise were analyzed with a mixed-effects analysis (for complete blood counts).

### Study approval.

This trial was conducted under the approval of a United States Food and Drug Administration (FDA) Investigational New Drug application (IND 142734) and the Johns Hopkins University IRB (IRB00223615). Written informed consent was obtained from all patients prior to participation in study procedures.

### Data availability.

The data that support the findings of this study including the supplemental materials are available in a separate supporting data file available online. Further study-related human subject data is available from the corresponding author upon request and will be deidentified before sharing.

## Author contributions

The trial was conceived of and designed by MCD and BSB. RVS and MCD oversaw the conduct of the trial and took lead roles in all aspects of study visits and patient care. Patient recruitment and screening was done by RVS. RAW assisted in patient recruitment. Collection of data was performed by MCD, RVS, CD, and DWZM. Data analyses were conducted by MCD and DV. The manuscript was written by RVS and MCD. All of the authors had access to the data and vouch for its completeness and accuracy and for fidelity to the protocol. All contributing authors were involved in review and final approval of the manuscript.

## Supplementary Material

Supplemental data

ICMJE disclosure forms

Supporting data values

## Figures and Tables

**Table 1:**
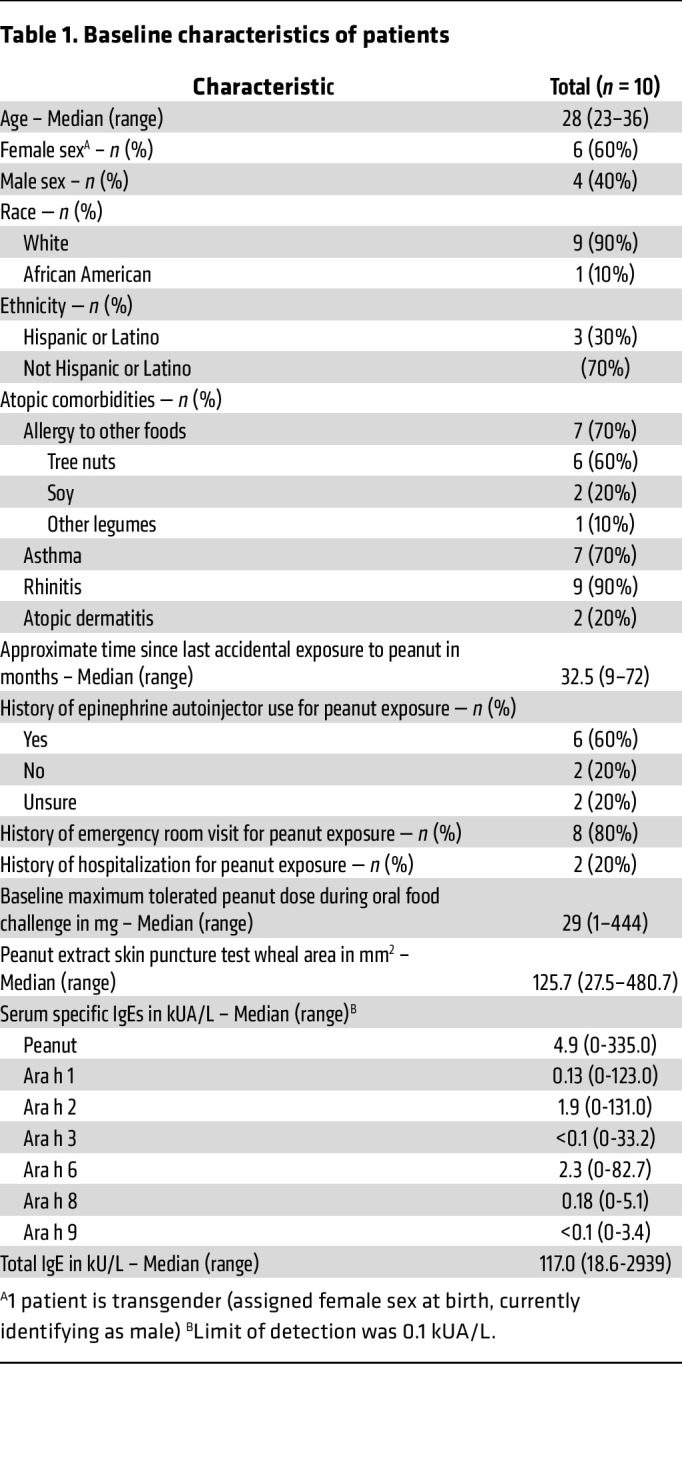
Baseline characteristics of patients

**Table 2:**
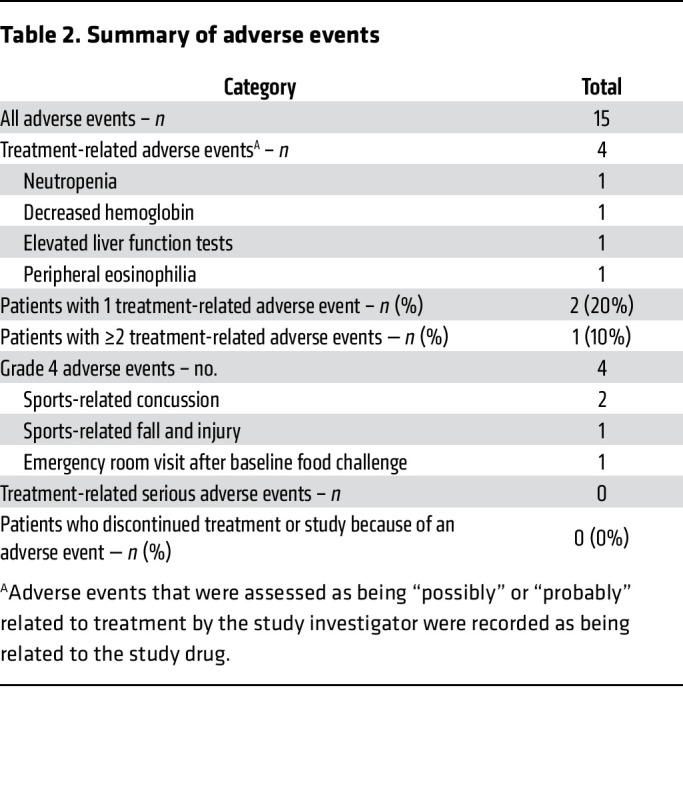
Summary of adverse events

## Version 1 06/29/2023

In-Press Preview

## Version 2 08/15/2023

Electronic publication
