# Supplementary material for: A phase II study of Bruton’s tyrosine kinase inhibition for the prevention of anaphylaxis
Source: J Clin Invest. 2023 Aug 15;133(16):e172335. doi: 10.1172/JCI172335 (PMC10425211; doi:10.1172/JCI172335)
Supplement: Supplemental data [file jci-133-172335-s070.pdf]

SUPPLEMENTAL INFORMATION

A phase 2 study of Bruton's tyrosine kinase inhibition for the prevention of anaphylaxis

This supplement contains the following items:

1. Supplemental Tables
2. Supplemental Figures
3. Study Protocol
4. Statistical Analysis Plan

SUPPLEMENTAL TABLES

Supplemental Table 1. Placebo-controlled, single-blinded, graded oral food challenge protocol

Food challenge dose	Form of food	Amount of food protein in dose (mg)	Cumulative peanut dose (mg protein)
**Placebo Challenge**			
1	Oat flour	30 mg	n/a
2	Oat flour	1 mg	n/a
3	Oat flour	10 mg	n/a
**Peanut Challenge**			
4	Peanut flour	1 mg	1 mg
5	Peanut flour	3 mg	4 mg
6	Peanut flour	10 mg	14 mg
7	Peanut flour	30 mg	44 mg
8	Peanut flour	100 mg	144 mg
9	Peanut flour	300 mg	444 mg
10	Peanut flour	600 mg	1,044 mg
11	Peanut flour	1,000 mg	2,044 mg
12	Peanut flour	1,000 mg	3,044 mg
13	Peanut flour	1,000 mg	4,044 mg

Supplemental Table 2. Symptom scoring system during oral food challenges

Symptom	Score
**I. Skin**	
**A. Pruritus**	
Absent	0
Mild: occasional scratching	1
Moderate: scratching continuously for >2 minutes at a time	2
Severe: hard, continuous scratching; excoriations	3
**B. Urticaria/Angioedema**	
Absent	0
Mild: <3 hives or mild lip edema	1
Moderate: <10 hives but >3; significant lip/face edema	2
Severe: generalized involvement	3
**C. Rash**	
Absent	0
Mild: few areas of faint erythema	1
Moderate: areas of erythema	2
Severe: generalized marked erythema (>50%)	3
**II. Upper Respiratory**	
**A. Sneezing/Itching**	
Absent	0
Mild: rare bursts; occasional sniffing	1
Moderate: bursts <10; intermittent rubbing of nose and/or eyes; frequent sniffing; mild conjunctival injection	2
Severe: continuous rubbing of nose and/or eyes; periocular swelling; long bursts of sneezing; persistent rhinorrhea; moderate-severe conjunctival injection	3
**B. Laryngeal**	
Absent	0
Mild: >3 discrete episodes of throat clearing or cough, or persistent throat tightness/pain; uvular erythema	1
Moderate: hoarseness, frequent dry cough; uvular edema (mild)	2
Severe: stridor; uvular edema (moderate-severe)	3
**III. Lower Respiratory**	
**A. Wheezing**	
Absent	0
Mild: expiratory wheezing to auscultation	1
Moderate: inspiratory and expiratory wheezing	2
Severe: use of accessory muscles; audible wheezing	3
**IV. Gastrointestinal**	
**A. Subjective Complaints**	
Absent	0
Mild: complaints of nausea or abdominal pain; itchy mouth/throat (mild)	1
Moderate: frequent complaints of nausea or pain with normal activity; itchy mouth/throat (moderate-severe)	2
Severe: notably distressed due to GI symptoms with decreased activity	3
**B. Objective Complaints**	
Absent	0
Mild: 1 episode of emesis or diarrhea	1
Moderate: 2-3 episodes of emesis or diarrhea or 1 of each	2
Severe: >3 episodes of emesis or diarrhea or 2 of each	3
**V. Cardiovascular/Neurologic**	
Normal heart rate or BP for age/baseline	0
Mild: subjective response (weak, dizzy), or tachycardia	1
Moderate: drop in blood pressure and/or >20% from baseline, or significant change in mental status	2
Severe: cardiovascular collapse, signs of impaired circulation; unconscious	3

Supplemental Table 3. Participant inclusion and exclusion criteria

**Inclusion Criteria**
History of immunoglobulin E (IgE)-mediated food allergy to peanut or tree nut
Positive skin puncture test to the trigger food (either peanut or tree nut)
Objective clinical reaction to 1,044 mg or less of the food allergen protein during baseline oral food challenge
Women of child bearing potential must agree to two forms of highly effective contraception (hormonal, device, or barrier method of birth control; abstinence) for the duration of study participation.
Ability to understand and the willingness to sign a written informed consent
Ability to clearly understand and speak English at an 8th grade reading level
**Exclusion Criteria**
Participants who have been on immunomodulatory therapies or oral corticosteroids within 1 month prior to enrollment
Participants with symptoms consistent with food reactions other than type 1 hypersensitivity
History of allergic reaction to acalabrutinib
History of idiopathic urticaria, dermatographism, idiopathic or unexplained anaphylaxis, or anaphylaxis (to foods or otherwise) resulting in intubation, prolonged hypotension, or neurological sequelae
History of cardiovascular disease
History of a bleeding disorder, or those currently taking blood thinners
History of stroke
History of gastrointestinal ulcer
History of cancer (other than skin cancer)
Positive HIV status or history of other immunodeficiency
Active or latent Hepatitis B or C infection based on laboratory testing
Currently pregnant or nursing
Current use of proton pump inhibitors (Note: participants currently receiving proton pump inhibitors who switch to H2-receptor antagonists or other antacids are eligible for enrollment to this study).
Active significant infection
Major surgical procedure within 28 days of enrollment
Uncontrolled autoimmune hemolytic anemia or idiopathic thrombocytopenic purpura
Difficulty swallowing oral medication, or significant gastrointestinal disease that would limit absorption of oral medication
Concurrent participation in another therapeutic clinical trial

Supplemental Table 4: Patient individual baseline characteristics and pretreatment oral food challenge reactions

Peanut IgE (kUA/L)	Ara h 1 IgE (kUA/L)	Ara h 2 IgE (kUA/L)	Ara h 3 IgE (kUA/L)	Ara h 6 IgE (kUA/L)	Ara h 8 IgE (kUA/L)	Ara h 9 IgE (kUA/L)	Serum tryptase (µg/L)	Tolerant peanut dose at baseline OFC	Symptoms of clinical reaction during baseline OFC
68.5	36.7	55.2	2.62	33.5	<0.1	1.18	6.3	444	Subjective: nausea, oropharyngeal pruritis, throat tightness<br>Objective: erythema of >50% BSA, conjunctival injection
<0.1	<0.1	<0.1	<0.1	<0.1	0.35	<0.1	2.5	14	Subjective: dizziness, nausea<br>Objective: erythema and urticaria (face, neck, back), periorbital edema
335	123	131	33.2	82.7	<0.1	<0.1	1.1	144	Subjective: nausea, abdominal pain, oropharyngeal and aural pruritis, lightheadedness<br>Objective: vomiting, conjunctival injection, facial erythema, wheezing
2.12	0.26	1.79	<0.1	1.65	<0.1	<0.1	2.7	14	Subjective: oral and aural pruritis<br>Objective: sneezing, cough, throat clearing, rhinorrhea, facial erythema, conjunctival injection
7.2	<0.1	1.92	0.15	2.91	5.07	3.38	9	44	Subjective: oropharyngeal pruritis, nausea, facial pruritis<br>Objective: erythema > 50% BSA, throat clearing, cough
3.22	<0.1	0.91	<0.1	0.12	2.78	<0.1	2.8	144	Subjective: oropharyngeal pruritis, throat tightness<br>Objective: uvular edema, wheezing
0.55	<0.1	0.14	<0.1	0.17	0.79	<0.1	4.6	1	Subjective: oropharyngeal and aural pruritis<br>Objective: throat clearing, uvular edema, facial erythema
0.72	0.32	0.26	<0.1	0.17	<0.1	<0.1	2.7	144	Subjective: oropharyngeal pruritis, throat tightness<br>Objective: throat clearing, erythema over shoulders, conjunctival injection, uvular edema
6.67	<0.1	4.82	<0.1	3.85	<0.1	2.61	3.2	14	Subjective: nausea, abdominal pain<br>Objective: conjunctival injection, uvular edema, erythema behind neck
31.7	8.92	19.1	2.16	4.53	2.25	<0.1	4.1	14	Subjective: nausea, oropharyngeal pruritis, throat tightness, neck pruritis<br>Objective: throat clearing, conjunctival injection, facial erythema, uvular edema

Patient ID	Age (yr)	Sex*	BMI (kg/m^2)	SPT wheal area to un-diluted peanut extract (mm^2)	Total IgE (kU/L)
**001**	36	M	29.4	138.2	329
**002**	34	M	27.3	27.5	50.5
**005**	32	F	32.8	255.3	802
**006**	26	F	39.2	113.1	18.6
**007**	34	F	48.7	75.4	2939
**008**	23	F	21	138.2	111
**009**	30	M	21.9	60.5	97
**010**	26	F	18.9	81.7	102
**011**	25	F	16.3	188.5	123
**013**	26	M	27	120.2	152

Abbreviations: BSA, body surface area; IgE, immunoglobulin E; OFC, oral food challenge; SPT, skin puncture test

*Sex assigned at birth

Supplemental Table 5. Summary of rescue medications administered during oral food challenges

Patient ID	Rescue medications administered during baseline oral food challenge	Rescue medications administered during acalabrutinib oral food challenge
001	Epinephrine 0.5 mL IM x 1 dose<br>Cetirizine 20 mg PO	None
002	Epinephrine 0.5 mL IM x 1 dose<br>Cetirizine 20 mg PO<br>Albuterol HFA 2 puffs via inhalation	None
005	Epinephrine 0.5 mL IM x 3 doses<br>Cetirizine 20 mg PO<br>Albuterol HFA 8 puffs via inhalation<br>Prednisone 60 mg PO<br>Supplemental oxygen (8 L)<br><br>In Emergency Department, received the following:<br>Diphenhydramine 25 mg PO<br>Famotidine 20 mg PO<br>Albuterol nebulization treatment 2.5 mg x 1 via inhalation	None
006	Epinephrine 0.5 mL IM x 1 dose<br>Cetirizine 20 mg PO	Epinephrine 0.5 mL IM x 1 dose<br>Cetirizine 20 mg PO<br>Albuterol HFA 4 puffs via inhalation
007	Epinephrine 0.5 mL IM x 1 dose<br>Cetirizine 20 mg PO	None
008	Epinephrine 0.5 mL IM x 1 dose<br>Cetirizine 20 mg PO<br>Albuterol HFA 2 puffs via inhalation	None
009	Epinephrine 0.5 mL IM x 1 dose<br>Cetirizine 20 mg PO	None
011	Epinephrine 0.5 mL IM x 1 dose<br>Cetirizine 20 mg PO<br>Albuterol HFA 8 puffs via inhalation	Epinephrine 0.5 mL IM x 1 dose<br>Cetirizine 20 mg PO<br>Albuterol HFA 4 puffs via inhalation
013	Epinephrine 0.5 mL IM x 1 dose<br>Cetirizine 40 mg PO<br>Albuterol HFA 1 puff via inhalation	Epinephrine 0.5 mL IM x 1 dose<br>Cetirizine 20 mg PO<br>Albuterol HFA 8 puffs via inhalation

Abbreviations: HFA, hydrofluoroalkane; IM, intramuscular; PO, per oral

Supplemental Table 6. Toxicity monitoring

Laboratory test	Baseline	During acalabrutinib therapy*	Follow-up	*p* =
White blood cell count (K/mm^3)	7.38 (1.58)	7.17 (2.11)	7.34 (2.76)	0.99
Hemoglobin (g/dL)	13.3 (1.60)	13.2 (1.44)	13.9 (1.25)	0.012
Platelet count (K/mm^3)	279 (105)	256 (106)	275 (122)	0.15
Absolute neutrophil count (K/mm^3)	4.56 (1.31)	3.76 (1.81)	4.27 (2.24)	0.27
Absolute lymphocyte count (K/mm^3)	1.99 (0.477)	2.43 (0.399)	2.21 (0.874)	0.008
Absolute monocyte count (K/mm^3)	0.502 (0.173)	0.540 (0.143)	0.504 (0.110)	0.43
Absolute eosinophil count (K/mm^3)	268 (119)	363 (257)	300 (155)	0.34
Absolute basophil count (K/mm^3)	50.0 (25.5)	48.0 (20.6)	48.5 (29.9)	0.97
Sodium (mmol/L)	140 (1.64)	140 (1.48)	140 (1.58)	0.95
Potassium (mmol/L)	3.97 (0.295)	3.91 (0.331)	4.26 (0.508)	0.06
Chloride (mmol/L)	107 (1.93)	107 (2.67)	107 (2.17)	0.86
Glucose (mg/dL)	90.3 (6.69)	93.8 (10.3)	102 (29.7)	0.29
BUN (mg/dL)	11.9 (3.32)	12.9 (2.64)	14.2 (3.12)	0.09
Creatinine (mg/dL)	0.812 (0.141)	0.843 (0.170)	0.865 (0.110)	0.15
Calcium (mg/dL)	9.07 (0.320)	9.00 (0.350)	9.07 (0.211)	0.75
Total protein (g/dL)	7.21 (0.475)	7.10 (0.435)	7.36 (0.366)	0.27
Albumin (g/dL)	3.78 (0.503)	3.79 (0.264)	4.03 (0.467)	0.06
Alkaline phosphatase (U/L)	75.3 (20.3)	74.9 (23.6)	81.4 (29.5)	0.21
Aspartate aminotransferase (U/L)	19.9 (4.91)	19.8 (9.43)	21.4 (8.92)	0.73
Alanine aminotransferase (U/L)	28.5 (7.23)	28.2 (7.00)	37.2 (20.7)	0.16
Bilirubin (mg/dL)	0.500 (0.271)	0.630 (0.226)	0.530 (0.211)	0.14
Quantitative immunoglobulins (mg/dL)<br>IgG<br>IgA<br>IgM<br>IgE	 <br>1,057 (167)<br>213 (85.2)<br>105 (38.4)<br>472 (896)	 <br>1,058 (187)<br>210 (75.4)<br>105 (37.8)<br>470 (801)	 <br>1,092 (174)<br>228 (82.6)<br>109 (41.0)<br>650 (1,015)	 <br>0.27<br>0.15<br>0.47<br>0.24

All values are expressed as mean (standard deviation). Data were analyzed using 1-way ANOVA.

*n = 9 for blood counts while on acalabrutinib therapy due to lab error (lost sample) for Patient 006. n = 10 for all other tests.

Supplemental Table 7. Total adverse events

Category	Total
Total adverse events – no.	15
Patients who experienced 1 adverse event – no (%)	1 (10%)
Patients who experienced ≥2 adverse events – no (%)	4 (40%)
Grade 1 adverse events – no.	
Anemia	1
COVID-19 infection (asymptomatic)	1
Eosinophilia	1
Elevated liver function tests (Alk Phos, AST, and ALT)	1
Hyperglycemia	1
Hypoalbuminemia*	1
Neutrophilia	1
Grade 2 adverse events – no.	
Anemia*	1
Hyperglycemia	1
Hypokalemia*	1
Neutropenia	1
Grade 4 adverse events – no.	
Emergency-room visit after baseline food challenge	1
Sports-related fall and injury	1
Sports-related concussion	2

*Present at baseline prior to study procedures or drug, not worsened by acalabrutinib.

Abbreviations: Alk Phos, alkaline phosphatase; ALT, alanine aminotransferase; AST, aspartate aminotransferase

SUPPLEMENTAL FIGURES

Supplemental Figure 1. Skin testing to dilutions of peanut extract
Skin puncture test wheal area (in mm^2) to peanut extract dilutions as well as positive (histamine) and negative (saline) controls at patients' baseline (blue boxes), during treatment with acalabrutinib (orange boxes), and at follow-up (green boxes) are shown. Box plot midline represents the median, box depicts 25th and 75th percentiles, and whiskers depict range. Graph depicts data from all patients who completed treatment (n = 10). Data for histamine, saline, and undiluted peanut extract at baseline and acalabrutinib timepoints are also presented in Figure 3A of the main manuscript.

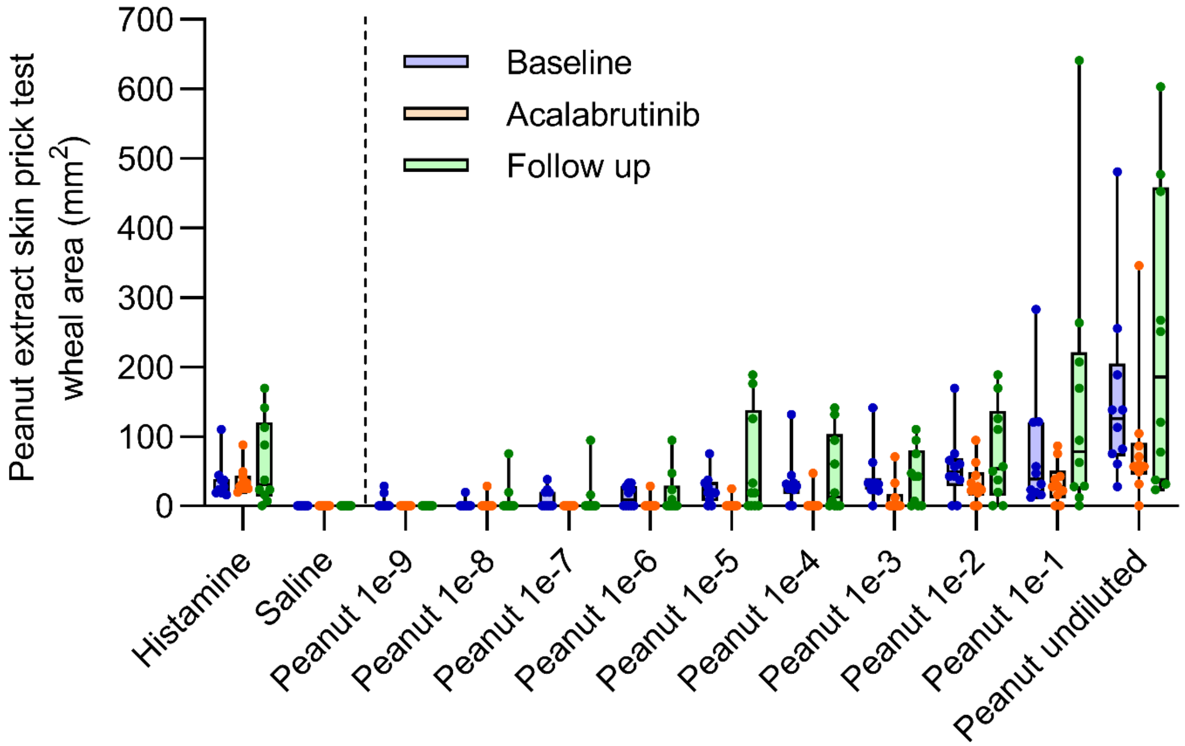

Supplemental Figure 2. Peanut specific IgE levels at all timepoints
Peanut and peanut-component specific IgE levels for all patients at baseline (blue boxes), during acalabrutinib treatment (orange boxes), and follow-up (green boxes) are shown. Box plot midline represents the median, box depicts 25th and 75th percentiles, and whiskers depict range. Data were analyzed using 2-way ANOVA. Graph depicts data from all patients who completed treatment (n = 10). Data for baseline and acalabrutinib are also presented in Figure 3D of the main manuscript.

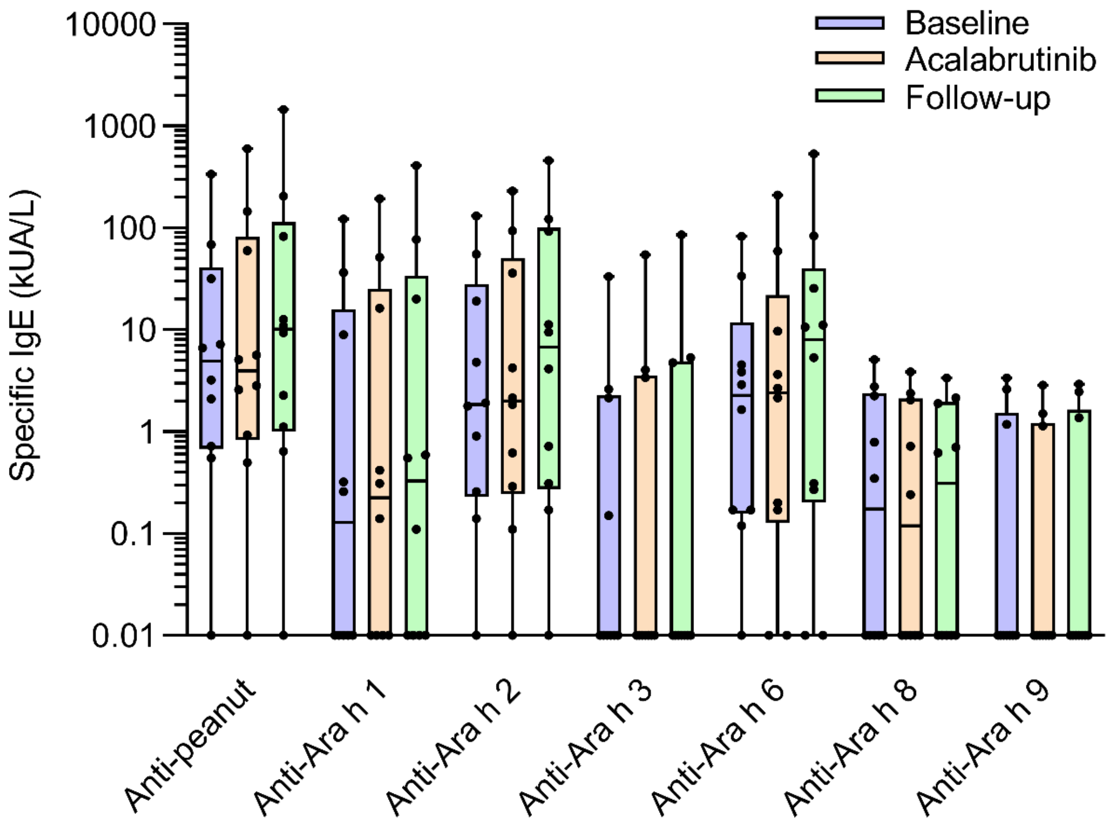

PROTOCOL

Study Title: **Preventing Life-Threatening Allergic Reactions with Acalabrutinib, an FDA-Approved BTK Inhibitor**

Principal Investigator: Melanie Dispenza, MD, PhD
Johns Hopkins University School of Medicine
Department of Medicine, Division of Allergy and Clinical Immunology
Johns Hopkins Asthma and Allergy Center
5501 Hopkins Bayview Circle, Room 3B.69
Baltimore, MD 21224
Phone: 410-550-1815
Fax: 410-550-1733
Email: mdispen1@jhmi.edu

Sub-Investigator(s): Donald MacGlashan, Jr, MD, PhD
Johns Hopkins University School of Medicine
Department of Medicine, Division of Allergy and Clinical Immunology

Biostatistician: Dhananjay Vaidya, MBBS, PhD, MPH
Associate Professor, Medicine
GeneSTAR Research Program
1830 E. Monument St, Room 8028
Baltimore, MD, 21287
Phone: 410-614-2440 / 410-550-5032
dvaidya1@jhmi.edu

Study Drug: Acalabrutinib (Brand name: Calquence®)

Protocol Number: MD12042018

Version: February 9, 2022

TABLE OF CONTENTS

LIST OF ABBREVIATIONS 1
STUDY SCHEMA 2
STUDY SUMMARY 2
1.0 BACKGROUND AND RATIONALE 4
1.1 Disease Background 4
1.2 Study Drug Background and Associated Known Toxicities 4
1.3 Rationale 4
2.0 STUDY OBJECTIVES 5
2.1 Primary Objective 5
2.2 Secondary Objectives 5
2.3 Endpoints 5
3.0 SUBJECT ELIGIBILITY 6
3.1 Inclusion Criteria 6
3.2 Exclusion Criteria 7
3.3 Study Stopping Criteria 8
3.4 Recruitment 8
4.0 TREATMENT PLAN 8
4.1 Treatment Dosage and Administration 8
4.2 Toxicities and Dosing Delays 9
4.3 Concomitant Medications/Treatments 9
4.4 Duration of Therapy 10
4.5 Duration of Follow Up 10
4.6 Subject Replacement 10
5.0 STUDY PROCEDURES 10
5.1 Screening/Baseline Procedures 10
5.2 Procedures During Treatment 11
5.3 Follow-up Procedures 13
5.4 Time and Events Table 13
5.5 Removal of Subjects from Study 13
6.0 RESPONSE CRITERIA 14
6.1 Safety/tolerability 14
6.2 Efficacy 14
7.0 ADVERSE EVENTS 14
7.1 Experimental Therapy 14
8.0 ADVERSE EVENT MONITORING 16
8.1 Definitions 17
8.2 Steps to Determine If an Adverse Event Requires Expedited Reporting 18
8.3 Reporting Requirements for Adverse Events 19
9.0 DRUG INFORMATION 20
9.1 Acalabrutinib 100 mg tablets 20
9.2 Return and Retention of Study Drug 20
10.0 STATISTICAL CONSIDERATIONS 21
10.1 Study Design/Study Endpoints 21
10.2 Sample Size and Accrual 21
11.0 STUDY MANAGEMENT 21
11.1 Conflict of Interest 21
11.2 Institutional Review Board (IRB) Approval and Consent 21
11.3 Data Management and Monitoring/Auditing 22
11.4 Adherence to the Protocol 22
11.5 Amendments to the Protocol 23
11.6 Confidentiality 23
11.7 Provisions to Protect the Privacy Interests of Subjects 23
11.8 Record Retention 23
11.9 Obligations of Investigators 24
12.0 REFERENCES 24

LIST OF ABBREVIATIONS

AE	Adverse Event
ALC	Absolute Lymphocyte Count
ALT	Alanine Aminotransferase
AST	Aspartate Aminotransferase
BAT	Basophil Activation Test
BTK	Bruton’s Tyrosine Kinase
BUN	Blood Urea Nitrogen
CBC	Complete Blood Count
CLL	Chronic Lymphocytic Leukemia
CMP	Comprehensive Metabolic Panel
CRU	Clinical Research Unit
SBPC	Single-blind Placebo-controlled
DLT	Dose-Limiting Toxicity
DSMB	Data and Safety Monitoring Board
EKG	Electrocardiogram
FC	Food Challenge
H&P	History & Physical Exam
HRPP	Human Research Protections Program
IgE	Immunoglobulin E
IV (or iv)	Intravenously
MCL	Mantle Cell Lymphoma
MTD	Maximum Tolerated Dose
OFC	Oral Food Challenge
PBMCs	Peripheral Blood Mononuclear Cells
p.o.	per os/by mouth/orally
PST	Peripheral Blood Testing
SAE	Serious Adverse Event
sIgE	Surface Immunoglobulin E
SGOT	Serum Glutamic Oxaloacetic Transaminase
SPGT	Serum Glutamic Pyruvic Transaminase
SPT	Skin Prick Test
WBC	White Blood Cells
WM	Waldenstrom’s Macroglobulineia

This is a phase II open label study involving the use of acalabrutinib to prevent food-induced anaphylaxis in adults with food allergy. Acalabrutinib (brand name Calquence®; manufacturer AstraZeneca [AZ]) is currently FDA approved for the treatment of mantle cell lymphoma (MCL). We propose to administer this approved drug to adults with food allergy to inhibit anaphylaxis.

STUDY SCHEMA

This is an open-label study designed to determine the ability of acalabrutinib to prevent signs and symptoms of anaphylaxis during a food challenge in food-allergic subjects.

At **Visit 1**, after undergoing informed consent, subjects will undergo procedures for assessment of baseline status and eligibility (clinical history and skin prick test [SPT] to peanut and/or tree nuts as well as histamine and saline SPT positive and negative controls, respectively). After eligibility is confirmed, subjects will undergo a single-blind, placebo-controlled graded food challenge (SBPCFC) of up to 4 g of peanut or tree nut protein, administered over escalating doses while being monitored in a clinical research unit (CRU) setting. Prior to the SBPCFC, 30 mL of peripheral blood will be collected for basophil activation testing (BAT), serum specific IgE to peanut/tree nuts and components, as well as toxicity lab testing monitoring. During the food challenge, subjects will be assessed for signs and symptoms of food-induced anaphylaxis between each dose per food challenge protocol, upon report of any symptoms and should any objective change in clinical status occur. Once a confirmed reaction occurs, the food challenge will be terminated and the reaction treated until it has resolved. Subjects will be discharged from the CRU no sooner than 2 hours after completion of the food challenge. They may be observed longer should a reaction occur at the investigator's discretion. Additional blood will be drawn at fixed intervals for biomarkers of anaphylaxis including mast cell and basophil microparticle analysis. This visit will establish each subject's baseline threshold dose for peanut/tree nut reactivity.

After a minimum of a two-week recovery period, subjects will be given acalabrutinib (four doses of 100 mg of acalabrutinib to be taken orally twice daily) and return for **Visit 2** to undergo repeat SPT, SBPCFC, and phlebotomy in an identical manner to Visit 1 to establish each subject's new threshold dose for peanut/tree nut reactivity while on acalabrutinib, which we hypothesize will be higher. At this point of the protocol, acalabrutinib dosing will be stopped. Follow up visits for up to 6 months after cessation of acalabrutinib will be conducted to repeat toxicity labs and skin testing.

At each visit, subjects will be monitored for toxicities, including CBC w/diff, CMP, and pregnancy testing. Prior to drug initiation and at the end of the trial, an EKG will be performed.

STUDY SUMMARY

Title	Preventing Life-Threatening Allergic Reactions with Acalabrutinib, an FDA-Approved BTK Inhibitor
Protocol Number	MD12042018
Phase	Phase 2
Methodology	Open label study to determine efficacy and safety of acalabrutinib for the treatment of food allergy
Study Duration	14 weeks
Study Center(s)	Single-center
Objectives	Primary: To determine the ability of acalabrutinib to 1) suppress food skin prick test size and 2) prevent food-induced anaphylaxis and/or shift the threshold dose of food at which a food allergic subject will react to a higher level.<br><br>Secondary: To determine if blood mast cell and basophil microparticles can be utilized as markers for anaphylaxis.
Number of Subjects	10

Diagnosis and Main Inclusion Criteria	Adults with history of food allergy to peanut and/or tree nut will be invited to participate.
Study Product(s), Dose, Route, Regimen	Acalabrutinib 100 mg, PO twice daily
Duration of administration	2 days
Reference therapy	There are currently no approved therapies for food allergy
Statistical Methodology	Primary efficacy endpoint: Change in highest tolerated dose of the food allergen. This endpoint will be analyzed by paired Student's *t* test.<br>Secondary efficacy endpoints will include:<br>1. change in severity of reaction during food challenge<br>2. change in skin prick test wheal size<br>3. change in BAT percent activation

1.0 BACKGROUND AND RATIONALE

1.1 Disease Background

Food allergy is a potentially life-threatening disease, and its prevalence continues to increase despite public health efforts (1, 2). Newer studies have shown that it is less likely that affected persons will develop natural tolerance over time, and food allergies can also develop in adulthood, so more adults now have potentially life-threatening food allergies. Severe and near fatal reactions can occur and can lead to devastating outcomes, including fatalities (3). Furthermore, the economic burden of childhood food allergy is nearly $25 billion per year, with as many as 200,000 emergency department visits annually (4). Despite this significant disease burden, there are currently no approved therapies for food allergy. Standard of care is constant vigilance in avoidance of allergen, and use of self-injected epinephrine after a reaction has occurred. Therefore, there is a large unmet need for new therapeutics to prevent food-induced anaphylaxis. FDA approved Bruton's tyrosine kinase (BTK) inhibitors such as ibrutinib, acalabrutinib and others under development (5, 6) may be able to fill this void and offer a therapy to these patients.

1.2 Study Drug Background and Associated Known Toxicities

Acalabrutinib is a twice daily, oral, highly selective, irreversible small-molecule inhibitor of BTK, a key component of B-cell receptor signaling (7, 8). BTK is also essential for FcεRI signaling and triggering of mast cells and basophils, the primary cells activated during anaphylaxis (9, 10). Acalabrutinib is currently FDA approved for the treatment of Mantle Cell Lymphoma (MCL).

Pharmacokinetic studies demonstrated the average bioavailability of acalabrutinib was 25%. Median time to peak acalabrutinib plasma concentrations (Tmax) was 0.75 hours. Following a single oral dose of 100 mg acalabrutinib, the median terminal elimination half-life ($t_{1/2}$) of acalabrutinib was 0.9 hours (range: 0.6 to 2.8). The $t_{1/2}$ of the active metabolite, ACP-5862, was 6.9 hours. Acalabrutinib is predominantly metabolized in the liver by CYP3A enzymes, and to a minor extent, by glutathione conjugation and amide hydrolysis, based on *in vitro* studies. Following administration of a single 100 mg radiolabeled acalabrutinib dose in healthy subjects, 84% of the dose was recovered in the feces and 12% of the dose was recovered in the urine, with less than 1% of the dose excreted as unchanged acalabrutinib.

In healthy volunteers, co-administration with CYP3A inhibitors increased Cmax, and therefore the drug will not be concomitantly administered with strong or moderate inhibitors of CYP3A. Among 1029 pooled patients from clinical trials (820 patients taking acalabrutinib monotherapy across 6 trials and 209 patients taking acalabrutinib plus obinutuzumab across 2 trials), most of whom (88%) were exposed to acalabrutinib for at least 6 months, adverse reactions in ≥30% of patients included anemia, neutropenia, upper respiratory tract infection, thrombocytopenia, headache, diarrhea, and musculoskeletal pain. Acalabrutinib is also pregnancy category D.

Notably, our prior work using ibrutinib (which may have a less favorable safety profile) in adults with food allergy demonstrated no toxicity or adverse effects from up to 7 days of treatment (11), suggesting that adverse effects from BTK inhibitors may be mitigated with short-term use rather than chronic treatment.

1.3 Rationale

There are currently no treatments for food allergy. IgE uniquely arms mast cells and basophils via their surface expression of FcεRI, and when cross-linked by allergens, causes mediator release that then results in an allergic reaction. Crosslinking of the receptor is known to activate a number of kinases. BTK is a key component of FcεRI signaling and activation of mast cells and

basophils. Since signaling mediated by FcεRI aggregation is thought to require the activity of BTK (8,9), MacGlashan et. al. was the first to test ibrutinib, a first generation BTK inhibitor, for its ability to prevent FcεRI activation of human basophils *in vitro* (9). Ibrutinib inhibited IgE-mediated CD63, CD203c and CD11b upregulation, and release of histamine, LTC4 and IL-4 as well as the cytosolic calcium response (IC_{90} of ≈50 nM). Ibrutinib did not inhibit basophil activation by fMLP or C5a and did not inhibit IL-13 release induced by IL-3, suggesting that BTK is required for IgE-mediated activation, but not IgE-independent activation, of human basophils (12). We recently completed an open label trial to test the hypothesis that ibrutinib would abrogate IgE-mediated BAT and food SPT responses in peanut and tree nut allergic adults (11). We found that 2 doses of ibrutinib significantly reduced SPT wheal and flare area (77 and 86% reductions, respectively; overall, 44% of SPTs became negative). Additional doses up to 7 days did not demonstrate further suppression compared to 2 days. Anti-IgE induced BAT became negative in all subjects after 2 doses and remained negative during treatment. The vast majority of SPTs and BAT returned to baseline within 1 week of cessation of ibrutinib. No toxicity from treatment was observed, and ibrutinib was well-tolerated by subjects.

We now propose to extend these promising findings in a subsequent open label study using acalabrutinib, a second generation BTK inhibitor, to investigate its ability to prevent clinical allergic reactions to oral food challenge. Because acalabrutinib is more selective for BTK with fewer off-target effects, it has a more favorable safety profile (13).

Peanut and tree nut allergies are among the most common adult food allergens, and ingestion reactions can often be the most severe and are the most likely to be life-threatening (1). Additionally, peanut and tree nut allergies are usually life-long. Young adults and adolescents have a higher rate of accidental exposure and mortality as a result of food allergies than younger children, so there is an unmet need for therapies directed at food allergy, especially in this population (14). Therefore, we have chosen peanut and tree nut allergies in an effort to investigate a treatment that has the potential to benefit the most individuals with a potentially severe allergy.

2.0 STUDY OBJECTIVES

2.1 Primary Objective

2.1.1 To determine if short term administration of acalabrutinib to adults with peanut or tree nut allergy will reduce allergen skin test reactions and raise oral challenge thresholds

2.2 Secondary Objectives

2.2.1 To describe frequency and severity of the adverse events associated with acalabrutinib when administered in a short course of 2 days

2.2.2 To determine if 2 days of acalabrutinib have any impact on blood levels of total and food-specific IgE, and numbers of circulating eosinophils and basophils.

2.3 Endpoints

Primary Objective 2.1.1: The highest dose of food allergen that is tolerated (i.e. does not cause an objective clinical reaction) during a SBPCFC both before and while taking acalabrutinib, as defined by objective clinical findings on physical exam before/after each dose of food ingested using a published graded scoring system (15). Secondary endpoints include change in severity of clinical reaction as defined above SPT wheal size as defined by a decrease in wheal and/or flare diameter of skin prick tests to peanut/tree

nut extracts, and percent activation of basophils from BAT as assessed by the percent of basophils with CD63 surface upregulation by flow cytometry.

Secondary Objective 2.2.1: Adverse events, including bleeding, cytopenias, hepatotoxicity, and renal toxicity will be monitored using laboratory testing and clinical exam as outlined in Section 4.2.

Secondary Objective 2.2.2: Change in blood levels of total and food-specific IgE, and numbers of circulating eosinophils and basophils as assessed by laboratory testing of peripheral blood samples.

Exploratory Objective: Peripheral blood will be analyzed for mast cell and basophil microparticles before, during, and after SBPCFC.

3.0 SUBJECT ELIGIBILITY

Subjects must meet all of the inclusion and exclusion criteria to be enrolled in the study. Study procedures may not begin until a subject has signed the informed consent form.

3.1 Inclusion Criteria

3.1.1 History of IgE-mediated food allergy to peanut or tree nut.

3.1.2 Male or female age ≥ 18 years.

3.1.3 Positive skin prick test to the trigger food(s), either peanut and/or tree nut(s).

3.1.4 Objective clinical reaction to 1,044 mg or less of the food allergen during baseline oral food challenge

3.1.5 Adequate organ and marrow function as defined below:

- leukocytes	≥ 3,000/mcL
- absolute neutrophil count	≥ 1,500/mcL
- hemoglobin	≥ 10 g/dL
- platelets	≥ 100,000/mcl
- total bilirubin	within normal institutional limits
- AST(SGOT)/ALT(SPGT)	within normal institutional limits
- Creatinine	within normal institutional limits

3.1.6 Women of child bearing potential must agree to two forms of highly effective contraception (hormonal, device, or barrier method of birth control; abstinence) prior to study entry, for the duration of study participation, and for 7 days following completion of acalabrutinib therapy. Should a woman become pregnant or suspect she is pregnant while participating in this study, she should inform the Principal Investigator and her treating physician immediately.

3.1.5.1 A female of child bearing potential is any woman (regardless of sexual orientation, having undergone a tubal ligation, or remaining celibate by choice) who meets the following criteria:

- Has not undergone a hysterectomy or bilateral oophorectomy; or
- Has not been naturally postmenopausal for at least 12 consecutive months (i.e., has had menses at any time in the preceding 12 consecutive months).

3.1.7 Ability to understand and the willingness to sign a written informed consent.

3.1.8 Ability to clearly understand and speak English at an 8th grade reading level. For safety reasons, subjects must speak English due to the anticipated need for clear and timely communication with investigators and the study team in emergency situations, since the investigators and study team are English speaking.

3.2 Exclusion Criteria

3.2.1 Subjects who have been on immunomodulatory therapies or oral corticosteroids within 1 month prior to study participation will be excluded, and those taking antihistamines must stop these drugs for one week prior to enrollment and must refrain from taking antihistamines during the duration of the study so as not to interfere with SPT responses or clinical responses during SBPCFC.

3.2.2 Subjects with symptoms consistent with food reactions other than type 1 hypersensitivity (such as atopic dermatitis, eosinophilic esophagitis and any other non-IgE-mediated food sensitivities) will be excluded.

3.2.3 History of allergic reactions to study drug or reactions attributed to compounds of similar chemical or biologic composition to acalabrutinib, including active product or excipients.

3.2.4 Clinically significant cardiovascular disease such as symptomatic arrhythmias, congestive heart failure, or myocardial infarction within 6 months of screening, or any Class 3 or 4 cardiac disease as defined by the New York Heart Association Functional Classification.

3.2.5 Psychiatric illness/social situations that would limit compliance with study requirements.

3.2.6 Subjects must not be pregnant or nursing due to the potential for congenital abnormalities and the potential of this regimen to harm nursing infants.

3.2.7 Subjects requiring or receiving vitamin K antagonists, those with active bleeding, and those with history of bleeding disorder (e.g. Von Willebrand's disease) or prothrombin time (PT)/INR or aPTT (in the absence of lupus anticoagulant) >2x upper limit of normal.

3.2.8 Subjects with history of idiopathic urticaria, dermatographism, idiopathic or unexplained anaphylaxis, or anaphylaxis (to foods or otherwise) resulting in intubation, prolonged hypotension, or neurological sequelae

3.2.9 Requires treatment with a strong cytochrome P450 3A4 (CYP3A4) inhibitor/inducer.or proton pump inhibitor. Note: Subjects receiving proton pump inhibitors who switch to H2-receptor antagonists or antacids are eligible for enrollment to this study.

3.2.10 Subjects found to have active or latent Hepatitis B or C infection based on laboratory testing. Subjects who are hepatitis B core antibody (anti-HBc) positive and who are hepatitis B surface antigen (HBsAg) negative will need to have a negative polymerase chain reaction (PCR) and must be willing to undergo DNA PCR testing during the study to be eligible. Those who are HBsAg positive or hepatitis B PCR positive will be excluded. Subjects who are hepatitis C antibody positive will need to have a negative PCR result to be eligible. Those who are hepatitis C PCR positive will be excluded.

3.2.11 Known history of infection with HIV or any active significant infection (eg, bacterial, viral, or fungal).

3.2.12 Major surgical procedure within 28 days of first dose of study drug. Note: If a subject had major surgery, they must have recovered adequately from any toxicity and/or

complications from the intervention before the first dose of study drug.

3.2.13 History of significant cerebrovascular disease/event, including stroke or intracranial hemorrhage, within 6 months before the first dose of study drug.

3.2.14 Presence of a gastrointestinal ulcer diagnosed by endoscopy within 3 months before screening.

3.2.15 Uncontrolled AIHA (autoimmune hemolytic anemia) or ITP (idiopathic thrombocytopenic purpura).

3.2.16 Prior malignancy (or any other malignancy requiring active treatment), except for adequately treated basal cell or squamous cell skin cancer, in situ cervical cancer.

3.2.17 Has diffculty with or is unable to swallow oral medication, or has significant gastrointestinal disease that would limit absorption of oral medication.

3.2.18 Concurrent participation in another therapeutic clinical trial.

3.3 Study Stopping Criteria

The study will be stopped if 2 or more subjects experience a serious adverse event, excluding adverse events that would be expected within the context of skin testing or oral food challenge, including medical treatment of clinical reactions during food challenge. Toxicities (section 4.2) and adverse events (section 7.0) are listed below. Monitoring is described in section 8.0.

3.4 Recruitment

Adults with a diagnosis of peanut or tree nut allergy by a board-certified allergist-immunologist will be identified as potential subjects from the allergy clinics and electronic medical record database search at the Johns Hopkins Hospital system. The treating physician will ask potential subjects for permission to be contacted by study personnel. Potential subjects will then be contacted by authorized study personnel and given time to review the study information and consider participation. If necessary, recruitment will be extended to include recruiting from local food allergy support groups and advertising on social media.

4.0 TREATMENT PLAN

4.1 Treatment Dosage and Administration

Prior studies in food-allergic subjects suggest that two standard (420 mg) doses (two days) of ibrutinib, an irreversible BTK inhibitor, are sufficient to completely inhibit BAT and suppress food SPT wheal size by an average of 77% (44% of all food SPTs became negative) (16). Additional doses of ibrutinib (up to 7 days) did not offer further suppression of SPTs, and BAT remained completely abolished for the duration of treatment. Therefore, we propose to extend these findings to acalabrutinib, another selective irreversible BTK inhibitor. Ten subjects will take acalabrutinib 100 mg twice daily by mouth (every 12 hours) for 2 days. Each subject will be enrolled for a minimum of 3 months (including the follow-up visit for toxicity monitoring).

Acalabrutinib is approved as a daily medication for another indication, and we propose to use the drug in adults only at its approved oral dosing of 100 mg twice daily for a maximum of two days. Dose limiting events include grade 3 or 4 hematologic events, grade 3 or 4 hepatotoxicity, AST or ALT >3 times normal range, creatinine > 2.5 times normal range. Dose-limiting toxicity (DLT) evaluation will be performed throughout the study.

The capsules should be swallowed intact with water. Subjects should not attempt to open capsules or dissolve them in water. Acalabrutinib can be taken with or without food.

If a dose is missed, it can be taken up to 3 hours after the scheduled time with a return to the normal schedule with the next dose. If it has been > 3 hours, the dose should not be taken and the subject should take the next dose at the scheduled time. The missed dose will not be made up and must be returned to the site at the next scheduled visit.

In healthy volunteers, co-administration with CYP3A inhibitors increased C_{max}, and therefore the drug will not be concomitantly administered with strong inhibitors or inducers of CYP3A.

4.2 Toxicities and Dosing Delays

Although toxicity is not expected given that subjects will only receive two days of drug, any subject who receives treatment on this protocol will be evaluable for toxicity. Each subject will be assessed for the development of toxicity according to the Time and Events table (Section 5.4).

At each visit, subjects will be monitored for toxicities. This study will follow the FDA Guidance For Industry ("Toxicity grading scale for healthy adult and adolescent volunteers enrolled in preventative vaccine clinical trials") grading system for adverse events (17). If a subject develops a grade 3 or 4 nausea, vomiting, diarrhea, hematologic toxicity, renal toxicity, or hepatotoxicity, or any other Grade 4 toxicity or unmanageable Grade 3 toxicity, the medication will be stopped. Protocols for specific toxicities are listed below.

Hematological Toxicities: ANC < 1,500/µL or platelets < 100,000/µL will result in discontinuation of protocol therapy.

Renal Toxicity: Creatinine will be monitored per protocol. Creatinine levels >2.5 times the normal limit will result in discontinuation of protocol therapy.

Hepatotoxicity: Grade 3 hepatotoxicity, or an increase in AST or ALT >3 times upper limit of normal, will result in discontinuation in protocol therapy.

4.3 Concomitant Medications/Treatments

At the systemic exposure levels expected in this study, acalabrutinib inhibition of CYP metabolism is not anticipated.

However, acalabrutinib is metabolized by CYP3A. Concomitant administration of acalabrutinib with a strong CYP3A and P-glycoprotein (P-gp) inhibitor, itraconazole increased exposure by approximately 5-fold. Conversely, concomitant administration of acalabrutinib with a strong CYP3A inducer, rifampin, decreased acalabrutinib exposure and could reduce efficacy. Consequently, the concomitant use of strong inhibitors/inducers of CYP3A should be avoided when possible.

If medically justified, subjects may be enrolled if such inhibitors or inducers can be discontinued or alternative drugs that do not affect these enzymes can be substituted within 7 days before first dose of study drug.

The effect of agents that reduce gastric acidity (eg, proton pump inhibitors or antacids) on acalabrutinib absorption was evaluated in a healthy volunteer study (ACE-HV-004). Results from this study indicate that subjects should avoid the use of calcium carbonate containing drugs or supplements for a period of at least 2 hours before and at least 2 hours after taking acalabrutinib. Use of omeprazole, esomeprazole, lansoprazole or any

other proton pump inhibitors while taking acalabrutinib is not recommended due to a potential decrease in study drug exposure. However, the decision to treat with proton-pump inhibitors during the study is at the investigator's discretion, with an understanding of the potential benefit to the subject's gastrointestinal condition and a potential risk of decreased exposure to acalabrutinib.

Although the effect of H2-receptor antagonists (such as famotidine or ranitidine) on acalabrutinib absorption has not been evaluated, if treatment with an H2-receptor antagonist is required, the H2-receptor antagonist should be taken approximately 2 hours after an acalabrutinib dose.

The following concomitant medications/treatments are not allowed: immunomodulatory therapies, oral corticosteroids within 1 month of study participation; those taking antihistamines must stop these drugs for one week prior to enrollment and must refrain from taking antihistamines during the duration of the study so as not to interfere with SPT responses; and any other prior investigational interventions in the past 6 months.

4.4 Duration of Therapy

Treatment will last for 2 days. Reasons the study medication may be discontinued include:

- Intercurrent illness that prevents further administration of treatment;
- Unacceptable adverse event(s);
- Subject decides to withdraw from the study; or
- General or specific changes in the subject's condition render the subject unacceptable for further treatment in the judgment of the investigator.

4.5 Duration of Follow Up

Subjects will be followed for 30 days after cessation of acalabrutinib treatment. Subjects will be removed from therapy when any of the criteria listed in Section 5.5 apply. The Principal Investigator will be notified. The reason for study removal and the date the subject was removed will be documented in the Case Report Form. Subjects removed from treatment for unacceptable adverse events will be followed until resolution or stabilization of the adverse event.

4.6 Subject Replacement

Subjects who withdraw voluntarily from the study, but were tolerating the drug will be replaced. Subjects who drop out or miss doses due to toxicity will not be replaced since these subjects will be considered to have experienced a dose limiting toxicity.

5.0 STUDY PROCEDURES

5.1 Screening/Baseline Procedures

Assessments performed exclusively to determine eligibility for this study will be done only after obtaining written informed consent. Assessments performed for clinical indications (not exclusively to determine study eligibility) may be used for baseline values even if the studies were done before informed consent was obtained.

All screening procedures must be performed within 14 days prior to baseline SBPCFC. The screening procedures include:

5.1.1 **Informed Consent**
Informed consent will be obtained per IRB and GCP guidelines.

5.1.2 **Medical history**
Complete medical and surgical history, including history of infections will be obtained.

5.1.3 **Demographics**
Age, gender, race, ethnicity will be obtained.

5.1.4 **Review subject eligibility criteria**
Subjects will be screened to ensure they meet inclusion and exclusion criteria, including history and prior laboratory testing consistent with a diagnosis of IgE-mediated food allergy.

5.1.5 **Review previous and concomitant medications**
Medications will be reviewed and documented.

5.1.6 **Physical exam including vital signs, height, and weight**
Vital signs (temperature, pulse, respirations, blood pressure), height, weight and basic full physical exam will be performed by study physicians.

5.1.7 **Adverse event assessment**
Baseline kidney, liver, and heart function will be assessed. This will include a CMP, CBC, Hepatitis B and C testing, and EKG. See section 6 for Adverse Event monitoring and reporting.

5.1.8 **Hematology**
Baseline CBC will be performed.

5.1.9 **Blood draw for correlative studies**
See Section 9.0 for details.

5.1.10 **Serum chemistries**
Comprehensive metabolic panel (CMP) to include: albumin, alkaline phosphatase, ALT/SGPT, AST/SGOT, BUN, creatinine, electrolytes (sodium, potassium, calcium, chloride, bicarbonate), glucose, and total bilirubin.

5.1.11 **Pregnancy test (for females of child bearing potential)**
See section 3.1.6.1 for definition.

5.1.12 **Immunoglobulin levels**
Total quantitative immunoglobulins (QUIGs), including IgG, IgA, IgM, and IgE, as well as serum specific IgE for peanut/tree nuts will be monitored

5.1.13 **Spirometry**
To be performed before SBPCFC if the patient has a history of asthma or exercise-induced bronchospasm.

5.2 Procedures During Treatment

5.2.1 **Screening/Baseline visit (Visit 1)**

Screening:

- Physical exam, vital signs
- Urine pregnancy test (if applicable)
- CBC w/diff
- CMP
- Hepatitis B and C testing
- Total immunoglobulin levels
- EKG
- Spirometry (only if the subject has a history of asthma)
- SPT to relevant foods, saline and histamine controls
- BAT

Procedures (to be done only if labs and EKG are within normal limits)

- Single-blind placebo controlled oral food challenge (SBPCFC) as below

- Microparticle analysis

All oral food challenges shall be performed as single (patient)-blinded, placebo-controlled food challenges (SBPCFC) according to standard published guidelines (18, 19). Placebo challenge and active challenge will be performed in that order on the same day if the subject has no symptoms during the first (placebo) challenge. If a reaction requiring treatment occurs during the placebo challenge, the next challenge should not be performed until the symptoms have subsided. Food challenge to peanut or tree nut protein will be performed according to standard operating procedure as previously published in the food allergy field (15).

<u>Actions to minimize risk of severe allergic reactions during oral food challenge):</u>
Prior to the study visit, subjects will be told to contact study staff about asthma and seasonal allergy symptoms, fever, acute infections including upper respiratory infections, rashes, or change in status of chronic medical conditions. Study visits will be rescheduled in the event that any of these occur. Study staff will contact subjects 1-3 days before study visits to inquire about recent symptoms.

All food challenges will be conducted in the Clinical Research Unit within the Johns Hopkins Bayview Hospital. Intravenous access will be obtained before the food challenge begins. Emergency medications (epinephrine, diphenhydramine, cetirizine, albuterol, ranitidine) will be immediately accessible in the subject’s room. All SBPCFCs will be conducted by trained personnel and supervised by a clinician with experience in performing SBPCFCs and trained in the treatment of anaphylaxis. Spirometry (assessment of pulmonary function) will be performed prior to the onset of the SBPCFC. Vital signs and pulse oximetry will be obtained at baseline and prior to each dose of the food allergen and q15 minutes thereafter with an observation period of at least 2 hours after the last dose is consumed (longer should a reaction occur). The SBPCFC will begin with a low dose, which will increase incrementally. At physician discretion, based upon history and possible subjective or very mild objective symptoms during a challenge, dosing intervals may be extended. The procedure will be stopped when clear objective symptoms are noted.

Subjects will be discharged after a period of observation appropriate to the nature of the symptoms. They will receive information regarding possible delayed manifestations, emergency contact information and home treatment. All subjects will be provided with an epinephrine auto-injector to take home (if they do not have one already) and instructions on how and when to use it.

After a minimum recovery period of 2 weeks, subjects will then take acalabrutinib as described above, and return on the second day of treatment for Visit 2 (see below).

5.2.2 **Visit 2**

- Physical exam, vital signs
- Urine pregnancy test (if applicable)
- CBC w/diff
- CMP
- Total immunoglobulin levels
- EKG
- Spirometry (only if the subject has a history of asthma)
- SPT to relevant foods, saline and histamine controls
- BAT
- SBPCFC (performed as described above)
- Microparticle analysis

5.3 Follow-up Procedures

Toxicity monitoring (including hematology, serum chemistries, QUIGs, ssIgE) and other study procedures will be repeated at 3 months after cessation of acalabrutinib; if at this time toxicity related lab results are not yet back to baseline, they will be repeated at 6 months.

- Physical exam, vital signs
- CBC w/diff
- CMP
- Total immunoglobulin levels
- SPT to relevant foods, saline and histamine controls
- BAT

5.4 Time and Events Table

**Visit**	1	2	3
	Screening / Day 0	Day 2	Follow up (30 days after Visit 2)
Informed Consent	X		
Skin prick testing	X	X	X
Basophil activation test	X	X	X
SBPC food challenge	X	X	
History and PE	X	X	X
Toxicity (include DLT) Evaluations	X	X	X
CBC	X	X	X
CMP	X	X	X
EKG	X	X	
Urine pregnancy test[b]	X	X	X
Microparticle analysis	X	X	X

[b] Females of child-bearing potential only

5.5 Removal of Subjects from Study

Subjects can be taken off the study treatment and/or study at any time at their own request, or they may be withdrawn at the discretion of the investigator for safety, behavioral or administrative reasons. The reason(s) for discontinuation will be documented and may include:

5.5.1 Subject voluntarily withdraws from treatment (follow-up permitted);
5.5.2 Subject withdraws consent (termination of treatment and follow-up);
5.5.3 Subject is unable to comply with protocol requirements;
5.5.4 Subject experiences toxicity that makes continuation in the protocol unsafe;
5.5.5 Treating physician judges continuation on the study would not be in the subject's best interest;

5.5.6 Subject becomes pregnant (pregnancy to be reported along same timelines as a serious adverse event); or

5.5.7 Lost to follow-up. If a research subject cannot be located after a period of 2 months, the subject may be considered “lost to follow-up.” All attempts to contact the subject during the 2 months must be documented.

6.0 RESPONSE CRITERIA

6.1 Safety/tolerability

Analyses will be performed for all subjects having received at least one dose of study drug. CBC, CMP, EKG will be performed on all subjects to monitor for toxicities and safety.

6.2 Efficacy

Increase in quantity of food that can be ingested on oral food challenge, decreased severity of reaction during oral food challenge, decrease in skin prick test responses and decreased basophil activation will be used to assess efficacy of the treatment, which will be assessed as described in Section 2.3.

7.0 ADVERSE EVENTS

7.1 Risks Associated with Experimental Therapy

The following summarizes the experience with acalabrutinib in hematologic cancer studies. For the most recent safety update, please refer to the current Study Agent Prescribing Information. (This drug is currently FDA approved for alternate indications. Sections below are from the current package insert).

7.1.1 Contraindications

No contraindications are known for acalabrutinib.

7.1.2 Warnings and Precautions for Use

As the subjects enrolled in this study will be generally healthy except for food allergy or other allergic disorders, and on acalabrutinib for short duration (2 days), we expect adverse events to be milder and less frequent, if at all, compared to prior studies of acalabrutinib in cancer trials. However, for completeness, adverse events as documented with chronic use are detailed below.

7.1.2.1 Hemorrhage

Serious hemorrhagic events, including central nervous system, respiratory, and gastrointestinal hemorrhage, have been reported in clinical trials with acalabrutinib; some of these bleeding events resulted in fatal outcomes. Grade 3 or higher bleeding events, including gastrointestinal, intracranial, and epistaxis have been reported in 2% of patients. Overall, bleeding events including bruising and petechiae of any grade occurred in approximately 50% of patients with hematological malignancies.

The mechanism for hemorrhage is not well understood. Acalabrutinib may further increase the risk of hemorrhage in patients receiving antiplatelet or anticoagulant therapies and patients should be monitored for signs of bleeding.

Consider the benefit-risk of withholding acalabrutinib for 3-7 days pre- and post-surgery depending on the surgery and the risk of bleeding.

7.1.2.2 Infections

Serious infections (bacterial, viral or fungal), including fatal events and opportunistic infections, have been reported in clinical studies with acalabrutinib. The most frequently reported Grade 3 or 4 infection was pneumonia. Across the acalabrutinib clinical development program (including subjects treated with acalabrutinib in combination with other drugs), cases of hepatitis B virus (HBV) reactivation (resulting in liver failure and death in 1 case) and cases of progressive multifocal leukoencephalopathy have occurred in subjects with hematologic malignancies. Monitor patients for signs and symptoms of infection and treat as medically appropriate.

7.1.2.3 Cytopenias

Treatment-emergent Grade 3 or 4 cytopenias including neutropenia, anemia, and thrombocytopenia have occurred in clinical studies with acalabrutinib. Subjects should be closely monitored as appropriate.

7.1.2.4 Second Primary Malignancies

Events of second primary malignancies, including non-skin carcinomas, have been reported in clinical studies with acalabrutinib. The most frequently reported second primary malignancy was skin cancer. Advise protection from sun exposure.

7.1.2.5 Atrial Fibrillation and Flutter

Events of atrial fibrillation/flutter have been reported in clinical studies with acalabrutinib, particularly in subjects with cardiac risk factors, hypertension, diabetes mellitus, acute infections, and a previous history of atrial fibrillation.

The mechanism for atrial fibrillation is not well understood.

7.1.3 Embryo-Fetal Toxicity

Based on findings in animals, acalabrutinib can cause fetal harm when administered to a pregnant woman. In female rats, acalabrutinib administered orally ≥ 100 mg/kg/day produced maternal toxicity and 100 mg/kg/day resulted in decreased fetal body weights and delayed skeletal ossification. Acalabrutinib and its active metabolite were present in the milk of lactating rats. Advise women to avoid becoming pregnant while taking acalabrutinib. If this drug is used during pregnancy or if the patient becomes pregnant while taking this drug, the patient will be apprised of the potential hazard to a fetus.

7.1.4 Interaction with other medications

At the systemic exposure levels expected in this study, acalabrutinib inhibition of CYP metabolism is not anticipated.

However, acalabrutinib is metabolized by CYP3A. Concomitant administration of acalabrutinib with a strong CYP3A and P-glycoprotein (P-gp) inhibitor, itraconazole increased exposure by approximately 5-fold. Conversely, concomitant administration of acalabrutinib with a strong CYP3A inducer, rifampin, decreased acalabrutinib exposure and could reduce efficacy. Consequently, the concomitant use of strong inhibitors/inducers of CYP3A be avoided when possible.

If medically justified, subjects may be enrolled if such inhibitors or inducers can be discontinued or alternative drugs that do not affect these enzymes can be substituted within 7 days before first dose of study drug. If a subject requires a strong CYP3A4 inhibitor while on study, the subject should be monitored closely for any potential toxicities.

The effect of agents that reduce gastric acidity (eg, proton pump inhibitors or antacids) on acalabrutinib absorption was evaluated in a healthy volunteer study (ACE-HV-004). Results from this study indicate that subjects should avoid the use of calcium carbonate containing drugs or supplements for a period of at least 2 hours before and at least 2 hours after taking acalabrutinib. Use of omeprazole, esomeprazole, lansoprazole or any other proton pump inhibitors while taking acalabrutinib is not recommended due to a potential decrease in study drug exposure. However, the decision to treat with proton-pump inhibitors during the study is at the investigator’s discretion, with an understanding of the potential benefit to the subject’s gastrointestinal condition and a potential risk of decreased exposure to acalabrutinib.

Although the effect of H2-receptor antagonists (such as famotidine or ranitidine) on acalabrutinib absorption has not been evaluated, if treatment with an H2-receptor antagonist is required, the H2-receptor antagonist should be taken approximately 2 hours after an acalabrutinib dose.

7.1.5 Adverse Reactions

As per package insert (21), adverse reactions are listed in Section 7.1.2. Among 1029 pooled patients from clinical trials (820 patients taking acalabrutinib monotherapy across 6 trials and 209 patients taking acalabrutinib plus obinutuzumab across 2 trials), most of whom (88%) were exposed to acalabrutinib for at least 6 months, adverse reactions in ≥30% of patients included anemia, neutropenia, upper respiratory tract infection, thrombocytopenia, headache, diarrhea, and musculoskeletal pain.

Of note, patients in the trial references took acalabrutinib for much longer duration that we propose in this study. Our prior work using ibrutinib (which has a less favorable side effect profile) in adults with food allergy demonstrated no toxicity or adverse effects from up to 7 days of treatment (11), suggesting that adverse effects from BTK inhibitors may be mitigated with short-term use rather than chronic treatment.

8.0 ADVERSE EVENT MONITORING

Adverse event data collection and reporting, which are required as part of every clinical trial, are done to ensure the safety of Subjects enrolled in the studies as well as those who will enroll in future studies using similar agents. Adverse events are reported in a routine manner at scheduled times during a trial. Additionally, certain adverse events must be reported in an expedited manner to allow for optimal monitoring of subject safety and care.

All subjects experiencing an adverse event, regardless of its relationship to study drug/device, will be monitored until:

- the adverse event resolves or the symptoms or signs that constitute the adverse event return to baseline;
- any abnormal laboratory values have returned to baseline;
- there is a satisfactory explanation other than the study drug for the changes observed; or

- death.

8.1 Definitions

8.1.1 Definition of Adverse Event

An adverse event (AE) is any untoward medical occurrence in a subject receiving study treatment and which does not necessarily have a causal relationship with this treatment. An AE can therefore be any unfavorable and unintended sign (including an abnormal laboratory finding), symptom, or disease temporally associated with the use of an experimental intervention, whether or not related to the intervention.

8.1.2 Severity of Adverse Events

This study will follow the FDA Guidance For Industry ("Toxicity grading scale for healthy adult and adolescent volunteers enrolled in preventative vaccine clinical trials") grading system for adverse events (17).

Briefly, the severity of an AE is graded as follows:

<u>Mild (grade 1):</u> the event causes discomfort without disruption of normal daily activities.

<u>Moderate (grade 2):</u> the event causes discomfort that affects normal daily activities.

<u>Severe (grade 3):</u> the event makes the subject unable to perform normal daily activities or significantly affects his/her clinical status.

<u>Life-threatening (grade 4):</u> the subject was at risk of death at the time of the event.

<u>Fatal (grade 5):</u> the event caused death.

8.1.3 Serious Adverse Events

A "serious" adverse event is defined in regulatory terminology as any untoward medical occurrence that:

8.1.3.1 Results in death.
8.1.3.2 Is life-threatening.
(the subject was at risk of death at the time of the event; it does not refer to an event that hypothetically might have caused death if it were more severe).
8.1.3.3 Requires in-subject hospitalization or prolongation of existing hospitalization for ≥ 24 hours.
8.1.3.4 Results in persistent or significant disability or incapacity.
8.1.3.5 Is a congenital anomaly/birth defect.
8.1.3.6 Is an important medical event.

Adverse Events (AEs) for malignant tumors reported during a study should generally be assessed as Serious AEs. If no other seriousness criteria apply, the 'Important Medical Event' criterion should be used. In certain situations, however, medical judgement on an individual event basis should be applied to clarify that the malignant tumor event should be assessed and reported as a Non-Serious

AE. For example, if the tumor is included as medical history and progression occurs during the study, but the progression does not change treatment and/or prognosis of the malignant tumor, the AE may not fulfill the attributes for being assessed as Serious, although reporting of the progression of the malignant tumor as an AE is valid and should occur. Also, some types of malignant tumors, which do not spread remotely after a routine treatment that does not require hospitalization, may be assessed as Non-Serious; examples include Stage 1 basal cell carcinoma and Stage 1A1 cervical cancer removed via cone biopsy.

Any event that does not meet the above criteria, but that in the judgment of the investigator jeopardizes the subject, may be considered for reporting as a serious adverse event. The event may require medical or surgical intervention to prevent one of the outcomes listed in the definition of “Serious Adverse Event“. For example: allergic bronchospasm requiring intensive treatment in an emergency room or at home; convulsions that may not result in hospitalization; development of drug abuse or drug dependency.

Pregnancy reporting: Female subjects of childbearing age reporting conception on study and up to 2 days after last dose must stop taking study drugs and must notify study staff immediately. Male subjects with partner conception on study must notify study staff immediately. Any pregnancy that occurs with the subject or with the partner of a treated subject as described above during this study will be reported, followed to conclusion, and the outcome reported. Any occurrences of pregnancy should be reported to the IRB and Regulatory Authorities per institutional and/or regulatory guidelines, and to AZ per contractual guidelines.

8.1.4 Adverse Events of Special Interest (AESI)

The following events are adverse events of special interest (AESIs) for subjects exposed to acalabrutinib, and must be reported to the sponsors expeditiously (see Section 6.4 for reporting instructions), irrespective of regulatory seriousness criteria or causality: Ventricular arrhythmias (e.g., ventricular extrasystoles, ventricular tachycardia, ventricular arrhythmia, ventricular fibrillation, etc).

8.2 Steps to Determine If an Adverse Event Requires Expedited Reporting

<u>Step 1</u>: Identify the type of adverse event.

<u>Step 2</u>: Grade the adverse event.

<u>Step 3</u>: Determine whether the adverse event is related to the protocol therapy
Attribution categories are as follows:

- Definite – The AE *is clearly related* to the study treatment.
- Probable – The AE *is likely related* to the study treatment.
- Possible – The AE *may be related* to the study treatment.
- Unrelated – The AE *is clearly NOT related* to the study treatment.

Note: This includes all events that occur within 30 days of the last dose of protocol treatment. Any event that occurs more than 30 days after the last dose of treatment and is attributed (possibly, probably, or definitely) to the agent(s) must also be reported accordingly.

<u>Step 4</u>: Determine the prior experience of the adverse event.
Expected events are those that have been previously identified as resulting from administration of the agent. An adverse event is considered unexpected, for expedited reporting purposes only, when either the type of event or the severity of the event is <u>not</u> listed in:

- the current known adverse events listed in the Agent Information Section of this protocol;
- the drug package insert;
- the current Investigator's Brochure

8.3 Reporting Requirements for Adverse Events

The Sponsor will submit appropriate reports to applicable local regulatory agencies and to ethics committees (ECs) as per local regulations. The Sponsor will notify AZ in parallel with submission to the IRB and concerned Regulatory Authority for Suspected Unexpected Serious Adverse Reactions (SUSARs) and within fifteen (15) calendar days of awareness for other SAEs or Special Situation Reports using individual unblinded or blinded case reports (Contingency SAE Report Form, Institution's Standard SAE Report Form, MedWatch, or CIOMS). New information will be submitted to AZ within the same time frame as initial reports.
Each recorded AE or SAE will be described by its diagnostic term, duration (eg, start and end dates), severity, regulatory seriousness criteria, if applicable, suspected relationship to the study drug, and any actions taken. If study drug is discontinued because of an SAE, this information must be included in the SAE report.

8.3.1 Expedited Reporting

- The Principal Investigator must be notified within 24 hours of learning of any serious adverse events, regardless of attribution, occurring during the study or within 30 days of the last administration of the study drug.
- The Johns Hopkins University IRB must be notified within 5 business days of any unanticipated problems involving risk to subjects or others (UPIRSO). The following events meet the definition of UPIRSO:
    1. Any serious event (injuries, side effects, deaths or other problems), which in the opinion of the Principal Investigator was unanticipated, involved risk to subjects or others, and was possibly related to the research procedures.
    2. Any serious accidental or unintentional change to the IRB-approved protocol that alters the level of risk.
    3. Any deviation from the protocol taken without prior IRB review to eliminate apparent immediate hazard to a research subject.
    4. Any new information (e.g., publication, safety monitoring report, updated sponsor safety report), interim result or other finding that indicates an unexpected change to the risk/benefit ratio for the research.
    5. Any breach in confidentiality that may involve risk to the subject or others.
    6. Any complaint of a subject that indicates an unanticipated risk or that cannot be resolved by the Principal Investigator.
- The FDA should be notified within 7 calendar days of any unexpected fatal or life-threatening adverse event with possible relationship to study drug, and 15 calendar days of any event that is considered: 1) serious, 2) unexpected, and 3) at least possibly related to study participation.
- The Sponsor will submit appropriate reports to applicable local regulatory agencies and to ethics committees (ECs) as per local regulations. The Sponsor will notify AZ in parallel with submission to the concerned Regulatory Authority for Suspected Unexpected Serious Adverse Reactions (SUSARs) and other SAEs as individual unblinded or blinded case reports (Sponsor's Standard SAE Report Form, MedWatch, or CIOMS) within fifteen (15) calendar days of awareness or quarterly SAE line listings. All

SAEs are to be submitted to the AstraZeneca Product Safety mailbox: AEMailboxClinicalTrialTCS@astrazeneca.com

- New information will be submitted to AZ within the same time frame as initial reports

8.3.2 Routine Reporting

All other adverse events - such as those that are expected, or are unlikely or definitely not related to the study participation - are to be reported annually to the FDA as part of regular data submission. These adverse events will still be reported to the sponsor on a monthly basis.

8.3.3 Stopping Rules

The study will be stopped if 2 or more subjects experience a serious adverse event, excluding adverse events that would be expected within the context of skin testing or oral food challenge, including medical treatment of clinical reactions during food challenge.

9.0 DRUG INFORMATION

9.1 Acalabrutinib 100 mg tablets

- Other names for the drug(s)/device: Calquence®
- Classification - type of agent/device: small molecule BTK inhibitor

Mode of action: Acalabrutinib forms a covalent bond with a cysteine residue in the BTK active site, leading to inhibition of BTK enzymatic activity. BTK is a signaling molecule of the B-cell antigen receptor (BCR) and cytokine receptor pathways. BTK's role in signaling through the B-cell surface receptors results in activation of pathways necessary for B-cell trafficking, chemotaxis, and adhesion. Nonclinical studies show that acalabrutinib inhibits malignant B-cell proliferation and survival *in vivo* as well as cell migration and substrate adhesion in vitro.

- Storage and stability: Acalabrutinib is stored at room temperature and the capsules are stable.
- Protocol dose: 100 mg twice daily for 2 days
- Preparation: as supplied from the manufacturer
- Route of administration for this study: by mouth
- Incompatibilities: N/A
- Availability: commercially available by prescription. Medication will be paid for through grant funding, and provided free of charge to subjects.
- Side effects: Most common side effects are anemia, neutropenia, upper respiratory tract infection, thrombocytopenia, headache, diarrhea, and musculoskeletal pain . Please refer to package insert for comprehensive list of side effects.
- Nursing implications: Acalabrutinib is category D. It should not be used in nursing mothers.

9.2 Return and Retention of Study Drug

Two bottles (120 pills) of study drug will be provided for free by its manufacturer, AZ, and obtained by the Research Pharmacy at Johns Hopkins University. Study drug will dispensed as needed, until next follow up. Therefore, we do not anticipate retention of study drug. However, if there is unused study drug, it will be disposed of in an appropriate manner.

Compliance will be assessed by subject diary and self-report.

10.0 STATISTICAL CONSIDERATIONS

As this is an open label study, each patient's responsiveness will be examined compared to themselves prior to initiation of treatment.

10.1 Study Design/Study Endpoints

This is an open label study of acalabrutinib in 10 food-allergic adults to determine the effect of pretreatment with acalabrutinib on food allergen reactivity in food-allergic subjects given acalabrutinib 100 mg twice daily for 2 days. The primary endpoint is the threshold dose of food at which a subject reacts before (at baseline) and after acalabrutinib therapy. Given the nature of the study, it will be stopped if 2 or more patients have a DLT.

10.2 Sample Size and Accrual

10 subjects allows for 80% power to detect a 3-fold increase (1.1 natural log units; i.e. 1 food dose escalation) in the threshold food dose assuming that difference in threshold from post to pre is normally distributed with a standard deviation of 1.1 natural log units using a paired *t* test with $p<0.05$ as an approximation (though non-parametric tests will be utilized in the final analysis if needed). Patients will be recruited from the allergy clinics at Johns Hopkins University with a diagnosis of peanut (or tree nut) allergy by a board-certified allergist-immunologist.

10.3 Data Analysis

As this is an open label trial, descriptive statistics will be used. If appropriate, the primary outcome variable of threshold dose will be log transformed, and a paired *t* test will be used. If assumptions for paired t test are violated, a Wilcoxon non-parametric sign test will be used. Each patient will be compared to him/herself prior to beginning treatment and at each time point in the study to assess SPT size, BAT response, and food challenge dose threshold and response. Similar methodologies will be used for analysis of adverse events.

11.0 STUDY MANAGEMENT

11.1 Conflict of Interest

Any investigator who has a conflict of interest with this study (patent ownership, royalties, or financial gain greater than the minimum allowable by their institution, etc.) must have the conflict reviewed Johns Hopkins University. All investigators will follow the University conflict of interest policy.

11.2 Institutional Review Board (IRB) Approval and Consent

It is expected that the IRB will have the proper representation and function in accordance with federally mandated regulations. The IRB should approve the consent form and protocol.

In obtaining and documenting informed consent, the investigator should comply with the applicable regulatory requirement(s), and should adhere to Good Clinical Practice (GCP) and to ethical principles that have their origin in the Declaration of Helsinki.

Before recruitment and enrollment onto this study, the subject will be given a full explanation of the study and will be given the opportunity to review the consent form. Each consent form must include all the relevant elements currently required by the FDA Regulations and local or state regulations. Once this essential information has been provided to the subject and the investigator is assured that the subject understands the implications of participating in the study, the subject will be asked to give consent to participate in the study by signing an IRB-approved consent form.

Prior to a subject's participation in the trial, the written informed consent form should be signed and personally dated by the subject and by the person who conducted the informed consent discussion.

11.3 Data Management and Monitoring/Auditing

As has been done previously for the phase 1 study of acalabrutinib (13), adverse events will be monitored throughout treatment and toxicities will be assessed using the National Cancer Institute Common Toxicity Criteria for AEs, with its latest version, currently version 4.03.

11.4 Adherence to the Protocol

Except for an emergency situation in which proper care for the protection, safety, and well-being of the study subject requires alternative treatment, the study shall be conducted exactly as described in the approved protocol.

11.4.1 Emergency Modifications

Investigators may implement a deviation from, or a change of, the protocol to eliminate an immediate hazard(s) to trial subjects without prior IRB approval. For any such emergency modification implemented, an IRB modification form must be completed within five (5) business days of making the change.

11.4.2 Other Protocol Deviations/Violations

All other planned deviations from the protocol must have prior approval by the Principal Investigator and the IRB. According to the IRB, a protocol deviation is any unplanned variance from an IRB approved protocol that:

- Is generally noted or recognized after it occurs
- Has no substantive effect on the risks to research participants
- Has no substantive effect on the scientific integrity of the research plan or the value of the data collected
- Did not result from willful or knowing misconduct on the part of the investigator(s).

An unplanned protocol variance is considered a violation if the variance:

- Has harmed or increased the risk of harm to one or more research participants.
- Has damaged the scientific integrity of the data collected for the study.
- Results from willful or knowing misconduct on the part of the investigator(s).
- Demonstrates serious or continuing noncompliance with federal regulations, State laws, or University policies.

If a deviation or violation occurs without prior approval from the Principal Investigator, please follow the guidelines below:

Protocol Deviations: Deviations should be summarized and reported to the IRB at the time of continuing review.

Protocol Violations: Study personnel should report violations within two (2) week of the investigator becoming aware of the event using the same IRB online mechanism used to report Unanticipated Problems.

11.5 Amendments to the Protocol

Should amendments to the protocol be required, the amendments will be originated and documented by the Principal Investigator. It should also be noted that when an amendment to the protocol substantially alters the study design or the potential risk to the subject, a revised consent form might be required. The written amendment, and if required the amended consent form, must be sent to the IRB for approval prior to implementation.

11.6 Confidentiality

The specimens will be coded and data will be stored in the Allergy-Immunology Research Labs, Mezzanine, McGaw Pavilion, or in locked file cabinets and/or on password protected computers. Only the study personnel will have access to the specimens and data generated. The PI and the study team members will be responsible for receipt or transmission of the data and specimens. The specimens will be maintained until exhausted or until the study ends. If there are remaining samples at the end of the study, any identifying information will be removed from the sample vial and the specimens will be treated with 10% bleach and discarded per standard practice. Unused portions of the specimens may be shared with other members of the University community. The original data will remain with the PI in the Allergy Division. No PHI will be shared.

11.7 Provisions to Protect the Privacy Interests of Subjects

Certain other people besides the PI and his research team may review the results of the study, such as administrative staff members from the Office of Research, Office of Research Integrity and members of the Institutional Review Board, and members of the Office for Human Research Protections. There is a slight risk that a subject’s privacy might be at risk. All of the above mentioned people in the respective capacities are obligated to protect a subject’s privacy and have training and certification in the safeguarding of the patient’s privacy. All data and results will be maintained in the Allergy-Immunology Research Labs, Mezzanine, McGaw Pavilion, in locked file cabinets and/or on password protected computers.

To help the subjects feel at ease, these safeguards are outlined in the consent form.

As far as information in medical records, only licensed medical personnel would have permission to access the information.

11.8 Record Retention

Study documentation includes all Case Report Forms, data correction forms or queries, source documents, Sponsor-Investigator correspondence, monitoring logs/letters, and regulatory documents (e.g., protocol and amendments, IRB correspondence and approval, signed subject consent forms).

Source documents include all recordings of observations or notations of clinical activities and all reports and records necessary for the evaluation and reconstruction of the clinical research study.

Government agency regulations and directives require that the study investigator must retain all study documentation pertaining to the conduct of a clinical trial. In the case of a study with a drug seeking regulatory approval and marketing, these documents shall be retained for at least two years after the last approval of marketing application in an International Conference on Harmonization (ICH) region. In all other cases, study documents should be kept on file until three years after the completion and final study report of this investigational study.

11.9 Obligations of Investigators

The Principal Investigator is responsible for the conduct of the clinical trial at the site in accordance with Title 21 of the Code of Federal Regulations and/or the Declaration of Helsinki. The Principal Investigator is responsible for personally overseeing the treatment of all study subjects. The Principal Investigator must assure that all study site personnel, including sub-investigators and other study staff members, adhere to the study protocol and all FDA/GCP/NCI regulations and guidelines regarding clinical trials both during and after study completion.

The Principal Investigator at each institution or site will be responsible for assuring that all the required data will be collected and entered onto the Case Report Forms. Periodically, monitoring visits will be conducted and the Principal Investigator will provide access to his/her original records to permit verification of proper entry of data. At the completion of the study, all case report forms will be reviewed by the Principal Investigator and will require his/her final signature to verify the accuracy of the data.

12.0 REFERENCES

1. Sicherer SH, Sampson HA. Food allergy: Epidemiology, pathogenesis, diagnosis, and treatment. The Journal of allergy and clinical immunology. 2014;133(2):291-307.
2. Branum AM, Lukacs SL. Food allergy among U.S. children: trends in prevalence and hospitalizations. NCHS Data Brief. 2008(10):1-8.
3. Sampson HA. Fatal food-induced anaphylaxis. Allergy. 1998;53(46 Suppl):125-30.
4. Gupta R, Holdford D, Bilaver L, Dyer A, Holl JL, Meltzer D. The economic impact of childhood food allergy in the United States. JAMA Pediatr. 2013;167(11):1026-31.
5. Burger JA. Bruton's tyrosine kinase (BTK) inhibitors in clinical trials. Curr Hematol Malig Rep. 2014;9(1):44-9.
6. Whang JA, Chang BY. Bruton's tyrosine kinase inhibitors for the treatment of rheumatoid arthritis. Drug Discov Today. 2014;19(8):1200-4.
7. Kuehn HS, Swindle EJ, Kim MS, Beaven MA, Metcalfe DD, Gilfillan AM. The phosphoinositide 3-kinase-dependent activation of Btk is required for optimal eicosanoid production and generation of reactive oxygen species in antigen-stimulated mast cells. Journal of immunology. 2008;181(11):7706-12.
8. MacGlashan D, Jr., Honigberg LA, Smith A, Buggy J, Schroeder JT. Inhibition of IgE-mediated secretion from human basophils with a highly selective Bruton's tyrosine kinase, Btk, inhibitor. Int Immunopharmacol. 2011;11(4):475-9.
9. Iyer AS, Morales JL, Huang W, Ojo F, Ning G, Wills E, et al. Absence of Tec family kinases interleukin-2 inducible T cell kinase (Itk) and Bruton's tyrosine kinase (Btk) severely impairs Fc epsilonRI-dependent mast cell responses. The Journal of biological chemistry. 2011;286(11):9503-13.
10. Wechsler JB, Schroeder HA, Byrne AJ, Chien KB, Bryce PJ. Anaphylactic responses to histamine in mice utilize both histamine receptors 1 and 2. Allergy. 2013;68(10):1338-40.

11. Dispenza MC, Pongracic JA, Singh AM, Bochner BS. Short-term ibrutinib therapy suppresses skin test responses and eliminates IgE-mediated basophil activation in adults with peanut or tree nut allergy. The Journal of allergy and clinical immunology. 2018;141(5):1914-6.
12. Regan JA, Cao Y, Dispenza MC, Ma S, Gordon LI, Petrich AM, et al. Ibrutinib, a Bruton's tyrosine kinase inhibitor used for treatment of lymphoproliferative disorders, eliminates both aeroallergen skin test and basophil activation test reactivity. The Journal of allergy and clinical immunology. 2017;140(3):875-9 e1.
13. Zhang Z, Zhang D, Liu Y, Yang D, Ran F, Wang ML, et al. Targeting Bruton's tyrosine kinase for the treatment of B cell associated malignancies and autoimmune diseases: Preclinical and clinical developments of small molecule inhibitors. Arch Pharm (Weinheim). 2018;351(7):e1700369.
14. Sampson HA, Aceves S, Bock SA, James J, Jones S, Lang D, et al. Food allergy: a practice parameter update-2014. The Journal of allergy and clinical immunology. 2014;134(5):1016-25 e43.
15. Sampson HA, Gerth van Wijk R, Bindslev-Jensen C, Sicherer S, Teuber SS, Burks AW, et al. Standardizing double-blind, placebo-controlled oral food challenges: American Academy of Allergy, Asthma & Immunology-European Academy of Allergy and Clinical Immunology PRACTALL consensus report. The Journal of allergy and clinical immunology. 2012;130(6):1260-74.
16. Dispenza MC, Pongracic JA, Singh AM, Bochner BS. Short-term ibrutinib therapy suppresses skin test responses and eliminates IgE-mediated basophil activation in adults with peanut or tree nut allergy. The Journal of allergy and clinical immunology. 2018.
17. Toxicity grading scale for healthy adult and adolescent volunteers enrolled in preventative vaccine clinical trials [Internet]. Center for Biologics Evaluation and Research, Food and Drug Administration. 2007. Available from: https://www.fda.gov/downloads/BiologicsBloodVaccines/ucm091977.
18. Nowak-Wegrzyn A, Assa'ad AH, Bahna SL, Bock SA, Sicherer SH, Teuber SS, et al. Work Group report: oral food challenge testing. The Journal of allergy and clinical immunology. 2009;123(6 Suppl):S365-83.
19. Bindslev-Jensen C, Ballmer-Weber BK, Bengtsson U, Blanco C, Ebner C, Hourihane J, et al. Standardization of food challenges in patients with immediate reactions to foods--position paper from the European Academy of Allergology and Clinical Immunology. Allergy. 2004;59(7):690-7.
20. Common Terminology Criteria for Adverse Events (CTCAE). (Version 4.0, May 28, 2009). National Institutes of Health, National Cancer Institute (<http://evs.nci.nih.gov/ftp1/CTCAE/CTCAE_4.03_2010-06-14_QuickReference_5x7.pdf>).
21. CALQUENCE Prescribing Information [Internet]. U.S. Food and Drug Administration. Revised: 11/2017. Available from: https://www.accessdata.fda.gov/drugsatfda_docs/label/2017/210259s000lbl.pdf.

Appendix 1: Pill Diary

MONTH ______________________

Sunday	Monday	Tuesday	Wednesday	Thursday	Friday	Saturday

The study coordinator will fill in the month and days.
Please document number of pills and time you took the ibrutinib.

STATISTICAL ANALYSIS PLAN

Study Design and Endpoints

This is an open label study of acalabrutinib in 10 food-allergic adults to determine the effect of pretreatment (100 mg twice daily for 4 doses total) with acalabrutinib on food allergen reactivity in food-allergic subjects. There are 3 planned study visits where endpoint assessments will be collected. The primary endpoint will be assessed at Visit 1 (baseline) and Visit 2 (after acalabrutinib). Secondary and exploratory endpoints will be assessed at Visits 1, 2, and 3 (follow-up).

Primary endpoint: Change in subjects' threshold dose of peanut protein to elicit an objective clinical reaction

Secondary endpoints:

1. Change in size of skin prick test wheal
2. Change in percent of ex vivo basophil activation to anti-IgE and peanut, as determined by CD63 expression

Safety considerations and toxicity monitoring: Laboratory testing (complete blood counts, serum chemistries) and electrocardiogram

Given the nature of the study, it will be stopped if >50% of patients have a DLT as described in the protocol.

Sample Size and Power Analysis

Sample size was calculated based on the primary endpoint, assuming that the difference in threshold from baseline to after acalabrutinib is normally distributed with a standard deviation of 1.1 natural log units. Therefore, 10 subjects allows for 80% power to detect a 3-fold increase (1.1 natural log units; i.e. 1 food dose escalation) in the threshold food dose, using a paired *t* test with $p<0.05$ as an approximation, though non-parametric tests will be utilized in the final analysis if needed.

Data Analysis

As this is an open label trial, each subject's responsiveness will be examined compared to themselves (at baseline) prior to initiation of treatment. Descriptive statistics will be used to analyze endpoints. The primary endpoint will be analyzed by log transformation and paired t test with p value < 0.05, if appropriate. If assumptions for paired t test are violated, a Wilcoxon non-parametric sign test will be used.

Secondary endpoints will be analyzed using ANOVA with repeated measures across all visits with p value < 0.05. For experiments with multiple dosing levels, we will first examine if there is a dose-level by treatment interaction. If there is an interaction, treatment effects in individual dosing levels will be examined. Non-parametric tests will be utilized in place of ANOVA if necessary. Corrections will be made for multiple comparisons, if applicable, using a Šídák or Bonferroni multiple comparisons correction with an alpha threshold of 0.05.

For safety endpoints, ANOVA will be used for analysis of individual laboratory values (blood counts or chemistries).
